# Longitudinal single-cell RNA sequencing of patient-derived primary cells reveals drug-induced infidelity in stem cell hierarchy

**DOI:** 10.1038/s41467-018-07261-3

**Published:** 2018-11-22

**Authors:** Ankur Sharma, Elaine Yiqun Cao, Vibhor Kumar, Xiaoqian Zhang, Hui Sun Leong, Angeline Mei Lin Wong, Neeraja Ramakrishnan, Muhammad Hakimullah, Hui Min Vivian Teo, Fui Teen Chong, Shumei Chia, Matan Thangavelu Thangavelu, Xue Lin Kwang, Ruta Gupta, Jonathan R. Clark, Giridharan Periyasamy, N. Gopalakrishna Iyer, Ramanuj DasGupta

**Affiliations:** 1 Genome Institute of Singapore, Cancer Therapeutics & Stratified Oncology 5, 60 Biopolis Street, #02-01 Genome, Singapore, 138672 Singapore; 2 National Cancer Centre Singapore, Cancer Therapeutics Research Laboratory, 11 Hospital Drive, 169610 Singapore, Singapore; 3 Tissue Pathology and Diagnostic Oncology, Royal Prince Alfred Hospital and University of Sydney, Sydney, NSW 2006 Australia; 4 Chris O’Brien Lifehouse and Sydney Head and Neck Cancer Institute, Sydney, NSW 2050 Australia; 5 Present Address: Department of Computational Biology, Indraprastha Institute of Information Technology, Okhla Industrial Estate, Phase III, New Delhi Delhi, 110020 India

## Abstract

Chemo-resistance is one of the major causes of cancer-related deaths. Here we used single-cell transcriptomics to investigate divergent modes of chemo-resistance in tumor cells. We observed that higher degree of phenotypic intra-tumor heterogeneity (ITH) favors selection of pre-existing drug-resistant cells, whereas phenotypically homogeneous cells engage covert epigenetic mechanisms to trans-differentiate under drug-selection. This adaptation was driven by selection-induced gain of H3K27ac marks on bivalently poised resistance-associated chromatin, and therefore not expressed in the treatment-naïve setting. Mechanistic interrogation of this phenomenon revealed that drug-induced adaptation was acquired upon the loss of stem factor *SOX2*, and a concomitant gain of *SOX9*. Strikingly we observed an enrichment of SOX9 at drug-induced H3K27ac sites, suggesting that tumor evolution could be driven by stem cell-switch-mediated epigenetic plasticity. Importantly, JQ1 mediated inhibition of *BRD4* could reverse drug-induced adaptation. These results provide mechanistic insights into the modes of therapy-induced cellular plasticity and underscore the use of epigenetic inhibitors in targeting tumor evolution.

## Abstract (web summary)

Drug resistance is one of the major causes of cancer-related deaths. Here, the authors using single cell RNA-seq of oral squamous cell carcinoma patient samples pre- and post-cisplatin treatment show that phenotypically homogenous cell populations display cell state plasticity, with poised chromatin marks at mesenchymal genes in epithelial cells, and that the loss of stem factor Sox2 but gain of Sox9 expression (with de novo gain of H3K27ac sites) is associated with drug-induced adaptation.

## Introduction

Tumors represent a complex ecosystem of cells residing in genetically and phenotypically diverse states^1,2^. The notion that tumors are clonal, and that they are constantly evolving under selection pressure was first proposed by Peter Nowell in the 1970s^1^. Since then intra-tumor heterogeneity (ITH) has been documented at various genetic and phenotypic levels. ITH driven diversity within cancer cell populations allow tumors to harbor specialized cells with tumor-initiating, drug-resistant and metastatic properties^3–6^. The selection and enrichment of pre-existing resistant cells has been shown to be one of the most common drivers of drug-resistance^7,8^. However, the maintenance of polyclonal tumors with arrays of specialized cells can be energetically expensive, and the extent of ITH can vary greatly across individual patient tumors. Therefore, how phenotypically homogeneous populations that do not display a high degree of ITH can evade the selection pressure of drug-treatment and metastasis, remains an important unanswered question. We hypothesized that homogeneous tumors may invoke alternative mechanisms, such as cellular reprogramming to acquire new phenotypic states, thereby generating phenotypic variation^9,10^. Cellular plasticity could thus provide homogeneous tumor populations with the selective advantage necessary to survive the onslaught of drug treatment, thereby promoting resistance. Notably, in the absence of any selection pressure the potential for cellular reprograming may remain camouflaged; however, it is revealed only upon the application of the selection pressure of chemotherapeutic drugs and/or metastasis.

Cancer stem-like cells (CSCs) have been shown to possess drug-resistant properties. The selection of such cells under therapeutic stress is a classic example of clonal selection. On the other hand, de-differentiation (where differentiated cells alter their transcriptional program to exhibit stem or progenitor-like properties), or trans-differentiation or “cellular-reprogramming” (a process of lineage infidelity) has been suggested to drive adaptive evolution. Cellular reprogramming has been associated with epigenetic plasticity of lineage-defining promoters/enhancers^11,12^. This plasticity provides the framework for either stochastic^13^ or deterministic (guided by lineage-defining pioneer factors)^11,14^ activation of gene regulatory programs leading to cell-state transitions. Therefore, it can be inferred that transcriptional plasticity in otherwise phenotypically homogeneous metastable cells could allow the emergence of new cell-types^15–17^. We hypothesized that stochastic or molecularly coordinated epigenetic reprogramming under selection pressure might play important functions in the acquisition of diverse new cell states (cellular reprogramming) within phenotypically homogeneous cell populations.

In this study, we sought to explore this hypothesis by investigating the survival strategies adopted by phenotypically homogeneous vs. heterogeneous tumors under the selection pressure of anti-cancer drugs, and metastasis. Patient-derived primary oral squamous cell carcinomas (OSCC) cell lines were used to model tumor evolution and its impact on CSC populations in conjunction with retrospective and prospective validation in clinical cohorts under similar selection pressure. OSCCs represent prototypical aggressive squamous cell carcinomas (SCC) with a 5-year survival rate of 40–50%^18^. Patients with OSCCs are generally treated with adjuvant cisplatin^19^. We used single-cell RNA-sequencing (scRNA-seq)^20^ to characterize the transcriptional dynamics encompassing four distinct stages of tumor evolution under the selection pressure of cisplatin, and metastatic dissemination. Using this approach, we were able to identify rare CSC populations within treatment-naive tumor cells with pre-existing resistance and metastasis associated transcriptional signatures. Surprisingly, in the absence of pre-existing phenotypic heterogeneity, we uncovered stress-induced trans-differentiation as a major driver of drug-resistance and metastasis. Mechanistically we demonstrate the function of pioneer factors in determining distinct stem cell states, implicating cellular heterogeneity and its utilization in driving resistant and metastatic phenotypes. Notably, temporal and functional interrogation of the epigenome provided insights into the interplay between these stem factors and chromatin remodelers (CRs) in ‘sensing’ and responding to cellular stress. Altogether, we demonstrate that pre-existing ITH leads to the selection of CSC cells under selection pressure. Stress-induced trans-differentiation on the other hand, drives adaptation of homogeneous tumor cell populations to convergent phenotypic states that are pre-determined by a poised bivalent epigenome.

## Results

### Drug-induced emergence of de novo cell state

Chemoresistance remains a major cause for tumor relapse, often resulting in metastatic disease and cancer-associated mortality. We sought to investigate the effect of cytotoxic treatment on modulating cell-states using specific phenotypic lineage markers in SCCs. To do this, we analyzed the expression of epithelial (E-cadherin; *ECAD*) and mesenchymal (Vimentin; *VIM*) characteristics in tumor tissues obtained from treatment naive and matched locally-recurrent samples from the patients with oral SCC (OSCC; *n* = 20). This cohort only included patients who developed local recurrence after treatment that included the use of cisplatin-based chemotherapy. Expression analysis suggested that the majority of tumors (16 of 20; 80%) develop resistance by selection of pre-existing clones as evidenced by the retention of either *ECAD* *+* epithelial or *VIM* *+* mesenchymal-like cell-states (Supplementary Figure 1a). On the contrary, 4 of 20 (~20%) patients displayed drug-induced plasticity, in that they developed resistance by de novo emergence of VIM expressing cells; thereby resulting in the emergence of mesenchymal-like cell states (Supplementary Figure 1b). We hypothesized that to survive the selection pressure of drug treatment, tumor cell populations could either (a) select pre-existing resistant cells from the repertoire of phenotypically heterogeneous sub-populations generated by overt ITH, and/or (b) adopt a new resistant cell-states/phenotype using ‘cellular plasticity and/or reprogramming’ as an adaptive mechanism.

**Fig. 1:**
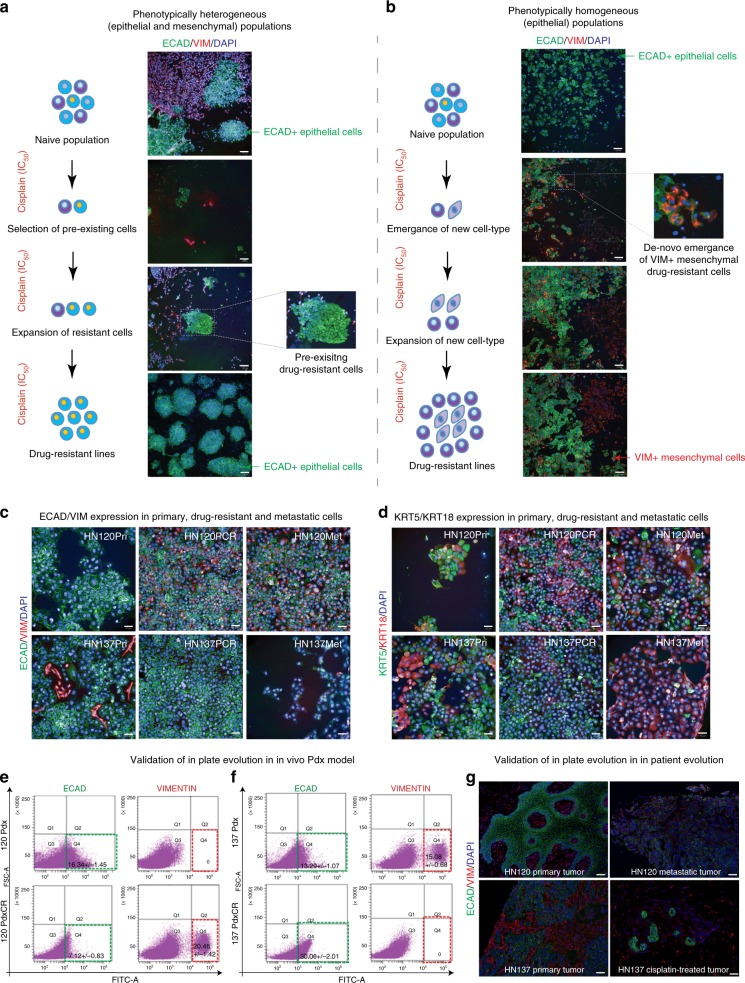
Modelling of drug-induced tumor evolution in vitro, in vivo and “in patients”. **a**, **b** Cisplatin treatment of PDPCs that are representative of phenotypically heterogeneous (HN137Pri) or homogeneous (HN120Pri) populations. Drug-induced selection of ECAD+ epithelial cells (green), and the elimination of Vim+ mesenchymal (red) cells, in HN137Pri model (**a**). De novo emergence of Vim + mesenchymal (red) cells within the phenotypically ECAD + epithelial (green) HN120Pri parental population (**b**) (*n* = 24 replicates per experimental condition). **c**, **d** Immunofluorescence-based characterization of naive and drug-resistant cells from HN120 and HN137 populations. Cells were stained for the expression of epithelial ECAD (in green) and mesenchymal VIM (in red) markers (**c**), as well as basal KRT5 (in green) and luminal KRT18 (in red) markers (**d**). **e**, **f** In vivo modelling of cisplatin-resistance in subcutaneous Pdx models of HN120Pri and HN137Pri cells (also see Supplementary Fig. 3a-g). Flow-cytometeric analysis of ECAD and VIMENTIN positive (VIM+) cells reveals a reduction of ECAD + population and the de novo gain of VIM + cells in the HN120 model (**e**). Loss of VIM + cells and an enrichment of ECAD + population is observed post-cisplatin selection in the HN137 model (**f**). The gating strategy for viable singlets is exemplified in Supplementary Figure 3a. **g** “In patient” validation of epithelial (ECAD+) to mesenchymal (VIM+) cell-state switch in HN120Met vs. HN120Pri patient tumor, and the selection of ECAD + epithelial cells with concomitant loss of VIM + mesenchymal cells in cisplatin treated HN137 patient recurrent (and thus resistant) tumor in the clinic. *n* = 24 replicates (**a**, **b**) and *n* = 3 replicates (**c**–**f**) per experimental condition). Scale bar = 100 μM (**a**, **b**, **g**), 50 μM (**c**–**d**)

To further interrogate the mechanistic basis for the two modes of resistance, we specifically selected two primary OSCC patient-derived cell lines (PDPCs), HN120Pri and HN137Pri, from our extensively characterized panel of PDPC models^21,22^. These patient-derived cells represented ideal models where HN137 displayed a mix of epithelial (ECAD+) and mesenchymal (VIM+) populations (Fig. 1a), while the HN120 line was comprised of phenotypically homogeneous population of ECAD+ cells (Fig. 1b). Thereafter, both populations were selected under cisplatin treatment followed by image-based analysis for ECAD and VIM expression every two weeks until resistance emerged. Approximately 100 single cells were seeded in each well of a 384-well plate and treated with the anti-cancer drug cisplatin (Fig. 1a, b). Each experimental condition was replicated 24 times to assess the reproducibility of the phenomenon. This setup allowed us to test two main outcomes: (i) if the frequency of resistant cells emerging out of the selection is >1:100 then resistant clones would be observed in every replica-well; otherwise we would expect to see resistant clones emerging only in a subset of the 24 wells; and (ii) a stochastic process may result in different phenotypes across wells while a deterministic process would lead to emergence and enrichment of resistant clones exhibiting similar cell-states/phenotype.

Within the first two weeks of cisplatin treatment, the heterogeneous HN137Pri cells demonstrated enrichment of ECAD+, and the gradual depletion of VIM+ cells (Fig. 1a). By the end of six weeks, the VIM+ cells were eliminated while a stable, homogeneous population of ECAD+ cells appeared to have been selected. This represents the classic example of overt ITH-mediated clonal selection. Surprisingly, for the HN120Pri model, which started off with an overtly homogeneous ECAD+ population, we observed the de novo emergence of VIM+ cells within two weeks of cisplatin treatment (Fig. 1b). Notably, a majority of the VIM+ cells also co-express the epithelial (ECAD) marker, thereby suggesting a drug-induced cell-state transition. These observations invoke the existence of covert mechanisms of drug-induced adaptation, which is phenotypically revealed only when cells experience a selection pressure. Continuous cisplatin treatment of HN120Pri cells resulted in a predominantly VIM+ population (Fig. 1b). Most strikingly, both selection of pre-existing clones in HN137 and cellular plasticity-mediated emergence of de novo cell states in HN120 were observed in all 24 experimental conditions, suggesting a deterministic process. Next, to ascertain if there were any rare (<1:10^6^ cells) pre-existing drug-resistant cells that could influence the resistant phenotype, we treated 1 × 10^7^ HN120Pri and HN137Pri cells with cisplatin for four months to generate resistant lines HN120PCR, HN120MCR, HN137PCR, and HN137MCR (Supplementary Figure 2a-c). This resulted in a similar phenotypic outcome to what was previously observed, corroborating the absence of clonal selection in HN120Pri, and instead suggesting the possibility of covert mechanisms that can drive cell-state transition and phenotypic variation (Fig. 1c, d).

### In vivo and ‘in patient’ validation of drug-resistant models

Since both cell intrinsic and extrinsic factors may contribute towards drug-resistance we assessed the effect of the microenvironment on drug-resistant phenotype by generating subcutaneous patient-derived xenograft (Pdx) models of HN120Pri and HN137Pri cells (Fig. 1e and Supplementary Figure 3). Pdx were treated with cisplatin for 3–4 weeks using standard protocols (see Methods); the tumors were subsequently harvested and characterized using markers against distinct cell states. Remarkably, similar to what we observed in our in vitro PDPC models, cisplatin treatment of HN137Pri Pdx also resulted in the selection of pre-existing ECAD+ epithelial cells with concomitant loss of VIM+ mesenchymal cells (Fig. 1e). Likewise, upon cisplatin treatment, the Pdx models of HN120Pri displayed de novo gain of the mesenchymal (VIM+) phenotype (Fig. 1f). Finally, even though Pdx models can provide stromal factors, they lack the immune component and more importantly, are devoid of the ‘human’ microenvironment. Therefore, we decided to compare our in vitro tumor-evolution models with the clinical phenotypes observed in matched patients. As shown in Fig. 1g, formalin-fixed-paraffin-embedded (FFPE) sections obtained from the recurrent, cisplatin-resistant tumor biopsy of patient HN137 demonstrated a striking gain of epithelial properties (ECAD+ cells) and a loss of mesenchymal phenotype. Due to unavailability of similar clinical drug-response model for HN120 we sought to validate PDPC associated cell-state transition in synchronous HN120Pri and HN120Met patient tumors. Both tumor and PDPC models displayed epithelial (ECAD+/VIM−) cells in primary tumor and gain-of mesenchymal (ECAD+/VIM+) properties in metastatic state (Fig. 1g). These results unequivocally demonstrate that PDPC models retain the cell-intrinsic properties of cancer cells found within the original tumors as well as respond to cisplatin treatment in a manner similar to what is observed in matched-patients.

### Functional characterization of de novo drug-resistant cell state

Drug-resistance and metastasis are often linked where resistant cells are known to acquire metastatic phenotypes and vice versa^23,24^. Therefore, in addition to primary HN120 and HN137 cisplatin-resistant cells, we also derived the resistant models of paired synchronous (lymph-node) metastasis from the same patients^21^. Both drug-resistant (HN137PCR and HN120PCR) and paired metastatic cells (HN137Met and HN120Met) were examined for the expression of basal (KRT5), luminal (KRT18), epithelial (ECAD), and mesenchymal (VIM) markers (Fig. 1c, d). HN120Pri cells exclusively expressed basal/epithelial markers while the drug-resistant counterpart (HN120PCR), and synchronous metastatic cells (HN120Met) expressed luminal/mesenchymal genes confirming cell-state transition (Fig. 1c, d, top panels). In sharp contrast, while the HN137Pri cells demonstrated heterogeneous populations of basal/epithelial and luminal/mesenchymal cells, their cisplatin-resistant derivative, HN137PCR, displayed a ‘selection’ of basal/epithelial cells (Fig. 1c, d, bottom panels). This phenotype was distinct from their synchronous metastatic counterparts (HN137Met) that displayed a strong luminal/mesenchymal phenotype (Fig. 1c, d). Functional assays corroborated these findings: in vitro scratch, trans-well migration and in vivo tail-vain injection assays revealed higher migratory and invasive properties of drug-resistant HN120PCR model while the HN137PCR cells demonstrated reduced metastatic properties, compared with their parental/treatment naive counterparts (Supplementary Figure 4a-c). Next, these cells were re-populated in the absence of cisplatin to model the ‘drug-holiday (DH)’ state (HN120PCRDH, HN120MCRDH, HN137PCRDH and HN137MCRDH)^10^. Interestingly, these drug-holiday cells remained refractory to cisplatin in long-term culture (>20 passages), (Supplementary Figure 2d) suggesting irreversible fixation of drug-resistant properties.

### Single-cell RNA-seq (scRNA-seq) to map the trajectories of drug-induced tumor evolution

**Fig. 2:**
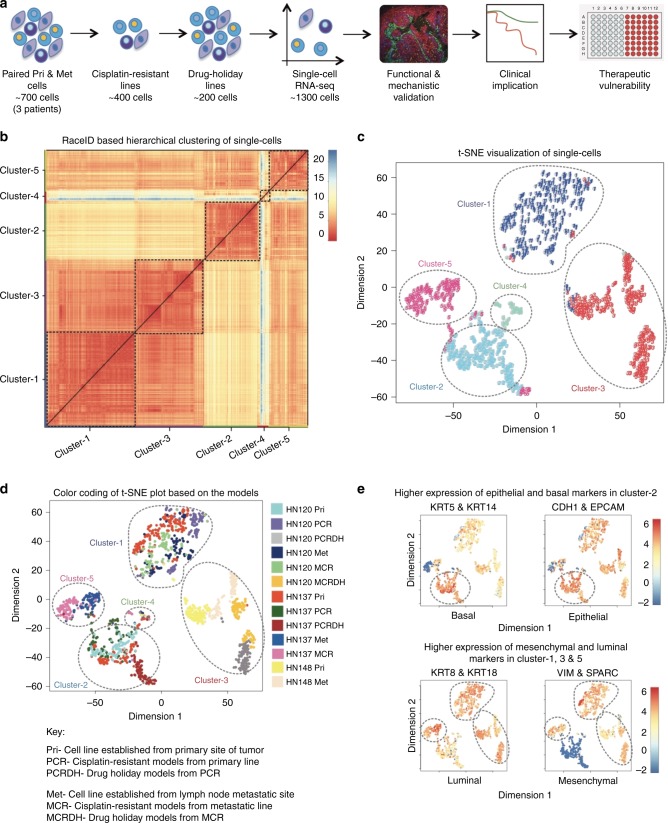
Single cell RNA-seq of naive, drug-resistant and drug-holiday cells from OSCCs. **a** Schematic representation of the generation of drug-resistant models and scRNA-seq workflow. **b** RaceID for Hierarchical k-means clustering of 1302 scRNA-seq libraries based on gene expression profiling identifies five major clusters. **c** t-SNE visualization of clusters identified from OSCC-PDPC scRNA-seq libraries. **d** Color-coding of t-SNE plot based on the identities of individual OSCC-PDPCs and their drug-resistant/holiday models. **e** Relative expression of genes associated with basal (KRT5/KRT14), luminal (KRT8/KRT18), epithelial (EPCAM/Ecad), and mesenchymal (VIM/SPARC) markers on tSNE plot suggesting that the clusters represent distinch phenotypic cell-states

In order to identify the origin of pre-existing or de novo emergence of drug-resistant states, we performed scRNA-seq on primary, metastatic, drug-resistant and drug-holiday models (Fig. 2a). In addition, as a reference control we also generated single-cell libraries from HN148 patient-derived cells that were inherently cisplatin-resistant (Supplementary Figure 2d). HN148 cells served as a prototypical model for treatment-naive intrinsically resistant state. In total, we generated transcriptome-wide expression profiles for 1302 single cells (1536 before quality control) representing variety of models (Supplementary Figure 5a and Supplementary Data 1). We hypothesized that rare ‘transcriptionally plastic’ epithelial cells^25^ could be present in the HN120Pri parental population that can reprogram to the mesenchymal-state observed in HN120PCR and HN120Met cells. Moreover, single cell transcriptomic profile of HN137Pri cells could reveal the existence of drug-resistant sub-clones of epithelial cells that were selected and enriched for in HN137PCR (Fig. 1a). To test these assumptions, we employed the recently developed RaceID algorithm^26^ specifically designed for the identification of rare sub-populations. Single-cell libraries were grouped based on gene expression correlation matrix followed by k-mean clustering. This resulted in the generation of five major clusters (Fig. 2b). We used t-distributed stochastic neighbor embedding (t-SNE)^27^ for dimension reduction (Fig. 2c, d). Cells from all patient models, and across different time points were found to be distributed across the five major clusters, suggesting minimal batch effect. Moreover, each cluster contained single cell libraries with comparable number of expressed genes, without demonstrating any bias for patient specific copy number variations (Supplementary Figure 5b-c). The specific gene expression patterns classified these clusters into two major phenotypic states, namely epithelial/basal and luminal/mesenchymal (Fig. 2e and Supplementary Data 1–2). Importantly, single cell gene expression clustering shows a consistent pattern with image-based validation of cell states (Fig. 1c, d and Supplementary Figure 5d). These results not only suggested the existence of a significant degree of intra- and inter-tumor heterogeneity, but also indicated association of both epithelial and mesenchymal cell-states with drug-resistance.

### Divergent modes of drug-resistance and metastatic evolution

**Fig. 3:**
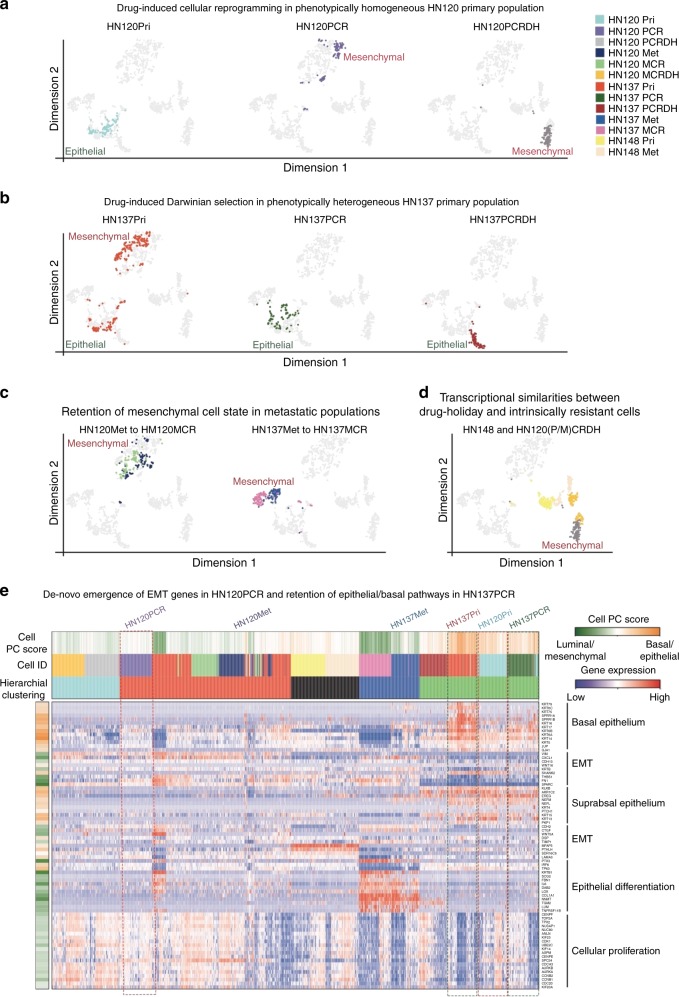
Divergent developmental trajectories of drug-resistance in OSCCs. **a**, **b** Distribution of naive primary cells and their drug-resistant/holiday models on tSNE plots for HN120 (**a**) and HN137 (**b**). Note the switching of HN120 cells between distinct phenotypic clusters as they evolve from epithelial HN120Pri (cyan) to mesenchymal resistant HN120PCR (purple) and drug-holiday HN120PCRDH (dark-grey) cell states (**a**). Also note the heterogeneity within the HN137Pri population (spread of ‘red’ cell between two clusters), and the selective retention of the epithelial cell state in the HN137PCR resistant (dark green) and HN137PCRDH holiday model (dark red) (**b**). **c** Visualization of HN120, and HN137 naive metastatic cells, as well as their drug-resistant counterparts on tSNE. **d** Clustering of HN120 primary and metastatic drug-holiday model with intrinsically resistant HN148 cells. **e** PCA (basal/luminal genes) based hierarchical clustering of OSCC-PDPCs and their drug-resistant/holiday models with PAGODA, note the retention of basal/epithelial properties in HN137PCR, while gain of mesenchymal gene-signature in HN120PCR

Corroborating the image-based analysis, HN120Pri cells clustered in a group (Fig. 3a) demonstrating relatively homogeneous transcriptome with higher expression of basal/epithelial markers. However, the matched drug-resistant (HN120PCR), drug-holiday (HN120PCRDH), and metastatic (HN120Met) cells acquired luminal/mesenchymal transcriptome (Fig. 3a). Importantly, we failed to detect pre-existence of rare drug-resistant and/or metastatic cells in the HN120Pri population, thereby alluding to the presence of covert drivers of cellular reprogramming in the treatment naive HN120Pri cells. On the other hand, HN137Pri cells displayed transcriptional heterogeneity that could be sub-classified into ‘basal/epithelial’ and ‘luminal/mesenchymal’ cell-types (Fig. 3b). Drug-resistant and holiday cells derived from HN137Pri displayed a ‘basal/epithelial’ signature suggesting cisplatin-induced selection of a pre-existing sub-population (Fig. 3b). The HN137Pri population also harbored pre-existing cells with mesenchymal signature that clustered with HN137Met, suggesting the existence of two distinct mesenchymal and cisplatin-resistant populations within the native cell population. Notably, cisplatin treatment of the metastatic cells from both HN120Met and HN137Met induced selection of pre-existing mesenchymal cells suggesting a ‘selective sweep’ in metastatic populations^28^ (Fig. 3c). The HN148Pri and HN148Met cells clustered with the HN120 drug-holiday cells (HN120PCRDH, HN120MCRDH) in a distinct luminal/mesenchymal group, emphasizing the similarities between cisplatin refractory drug-holiday models and intrinsically cisplatin-resistant cell-state (Fig. 3d). The distinct transcriptional profile of the HN120 drug-holiday cells compared to the drug-resistant models further corroborate the plasticity displayed by these cells. In contrast, the HN137 drug holiday cells largely retain the transcriptome of their parental and drug-resistant counterparts suggesting a selection-induced stable phenotype, similar to what has been recently reported in the case of vemurafinib resistance in *BRAF*-associated melanoma^25^.

Further validation by PCA-based analysis revealed that PC1 correlated with epithelial-cell differentiation while PC2 associated with epithelial-to-mesenchymal transition (EMT) (Supplementary Figure 6a-d and Supplementary Data 3). In addition, we determined the developmental pathways associated with drug-resistant models by single-cell differential expression^29^ (scde)-based pathway and gene-set over-dispersion analysis (PAGODA)^30^ (Supplementary Fig. 6e-f). Drug-resistant cells from HN120Pri demonstrated acquisition of EMT-like features, while those derived from HN137Pri, retained their epithelial-like phenotype (Fig. 3e). Taken together, three independent informatics-based analyses of the scRNA-seq data suggest that individual OSCC tumor cells can undergo divergent modes of evolution under the selection pressure of chemotherapeutic drugs and metastatic dissemination (Supplementary Figure 7).

### Slow-cycling epithelial cells contribute to selection of pre-existing clones

Our data revealed both selection of pre-existing clones (HN137) and cellular reprogramming (HN120) as mode of drug-resistance in OSCC. The CSC model of drug-resistance suggests that CSCs can escape chemotherapeutic treatment by the virtue of dormancy-induced slow cycling properties^31^. To examine this hypothesis, we determined the proliferative heterogeneity of epithelial (ECAD+) and mesenchymal (VIM+) cells in the HN120 and HN137 models. EdU based pulse-chase labeling experiments demonstrated a transition from epithelial (HN120Pri) to mesenchymal (HN120 PCR) cell-states without any substantial change in the cell-cycle status (Supplementary Fig. 8a,c). However, the HN137 model displayed selection of phenotypically quiescent epithelial cells while eliminating the fast dividing mesenchymal sub-population (Supplementary Fig. 8b, d). These results suggest that while HN137 model follows the classical CSC model of clonal selection of quiescent drug-resistant cells, the HN120 tumor cells follow an alternative, adaptive mode of resistance against cisplatin.

### Implication of ‘cell-of-origin’ in determining modes of drug-resistance

**Fig. 4:**
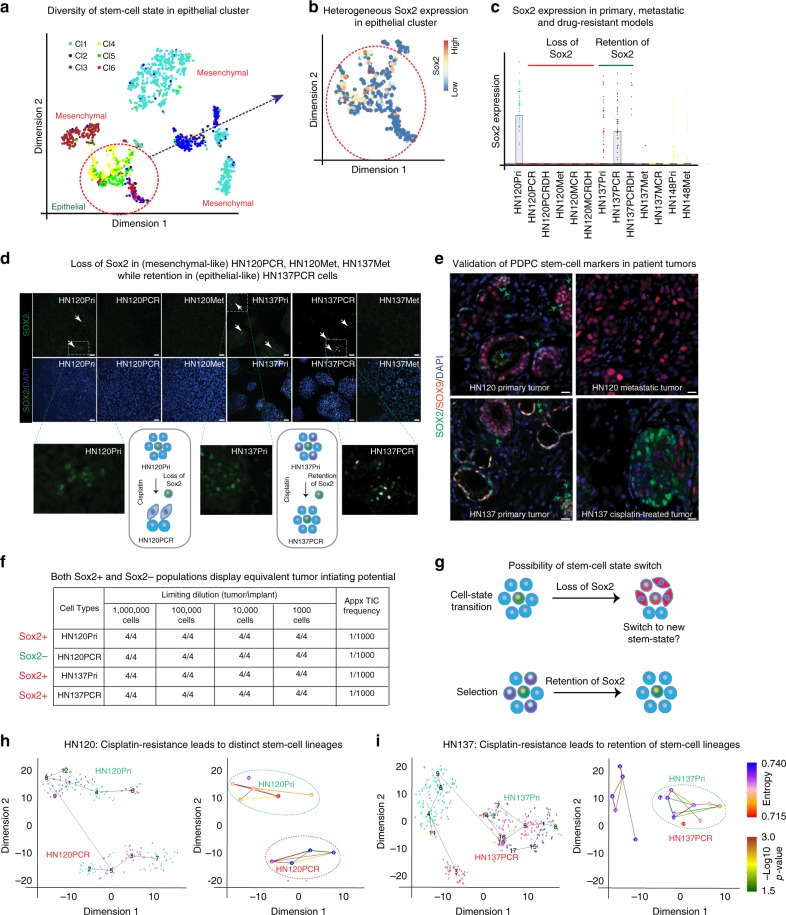
Drug-induced gain or loss of SOX2 determines the phenotypic identify of resistant cells. **a** Clustering of scRNA-seq libraries based on the expression of stemness genes followed by projection on original RaceID (depicted in Fig. 2d) tSNE plot. Cluster containing epithelial-like HN120Pri and HN137Pri cells (based on Fig. 2e) is marked with a red circle. Note the four sub-clusters within the epithelial-like cells based on the expression of stem cell markers. **b** Expression of SCC stem-like cell gene, SOX2, in a cluster consisting of epithelial-like HN120Pri and HN137Pri cells. **c** Beeswarm plot of SOX2 expression in different cell types from 1302 scRNA-seq libraries. **d** Immunofluorescence-based analysis of SOX2 (green) expression in HN120 and HN137 primary, drug-resistant, and metastatic models. Insets representing the magnified sections from respective immune-micrographs. **e** Validation of in vitro PDPC models in matched patients. Existence of SOX2 (green) and SOX9 (red) positive cells in HN120 primary patient tumor. Note the gain of SOX2+ (green) subpopulation in cisplatin treated HN137 patient tumor. Green arrows depict the sub-population of bona fide SOX2+ cells in primary tumors. **f** Limiting dilution assays (LDA) to assess the tumor-initiating potential of SOX2 expressing (HN120Pri, HN137Pri, and HN137PCR) vs. non-expressing HN120PCR cells (*n* = 4 mice per limiting dilution). **g** Proposed model for the stem cell-state switch from SOX2→SOX9 in HN120PCR and retention of SOX2 expressing phenotypic state in HN137PCR. **h**, **i** RaceID2 projection of the lineage tree of sensitive and resistant lines. HN120Pri and their cisplatin-resistant HN120PCR cells form two distinct lineage trees (**h**), while a common lineage-tree was observed in HN137Pri and their drug-resistant HN137PCR model (**i**). Scale bar = 50 μM (**d**, **e**)

Since our observations indicated distinct modes of drug-resistance in HN120 and HN137 cells we investigated whether these variations were linked to a “cell-of-origin”^32^. We performed hierarchal clustering of scRNA-seq data based on “stemness related” genes (Supplementary Data 4), which resulted in six major clusters (Fig. 4a). The epithelial cluster (based on RaceID) demonstrated the highest degree of heterogeneity for stem genes and could be further sub-divided into four clusters (Fig. 4a). Notably, the epithelial cells from HN120 and HN137 formed two distinct stem-clusters Supplementary Data 1). However, both groups expressed a stem gene *SOX2* (Fig. 4b). *SOX2* is a known oncogene expressed in a variety of SCCs, and is thought to be essential for the maintenance of tumor-initiating properties^33,34^. We observed cisplatin-induced selection of *SOX2*+ cells in HN137PCR while loss of these cells in HN120PCR (Fig. 4c). We further confirmed these observations by immunofluorescence that demonstrated the enrichment of SOX2+ cells in basal/epithelial HN137PCR cells. In sharp contrast, *SOX2* expression was undetectable in the mesenchymal-like HN120PCR, HN120Met, and HN137Met cells (Fig. 4d). Furthermore, we validated these results in the previously described Pdx models of HN120Pri and HN137Pri PDPCs treated with cisplatin. Similar to the in vitro models of cisplatin resistance, in vivo Pdx models mimicked the loss of SOX2+ with a concomitant gain of SOX9+ cells in the HN120 xenografts (Supplementary Figure 9a) and selection of SOX2+ cells in the case of HN137 (Supplementary Figure 9b). Most strikingly, we made identical observations in the matched patient samples from the clinic, particularly the selection of SOX2+ cells in cisplatin treated recurrent sample from patient HN137, and loss of SOX2+ alongwith the gain of SOX9+ expressing cells in patient HN120’s lymph-node metastatic tumor. Altogether these results unequivocally demonstrate that insights revealed from scRNA-seq based longitudinal analyses of PDPCs closely reflect the evolutionary trajectories of patient tumors in the clinic.

Interestingly, our results also indicate a putative function for *SOX2* in the retention of the epithelial state in SCCs. Surprisingly however the data generated with the HN120PCR and HN120Met models indicated that the loss of *SOX2* could also result in the acquisition of cisplatin-resistance, which is contrary to the notion of *SOX2* expression alone being sufficient in driving stem-like resistant cell states. Therefore next, we analyzed the genes that were differentially expressed in the *SOX2*+ cells between HN120Pri and HN137Pri cells. Intriguingly, the *SOX2*+ HN120Pri cells exhibited higher expression of the chromatin modifiers suggesting the possibility for distinct epigenetic states of the cell-of-origin within different SCC tumors (Supplementary Figure 9c). We also performed limiting dilution assay (LDA) to assess the tumor-initiating capabilities of *SOX2*+ positive cells from HN120Pri, HN137Pri, and HN137PCR, as well as from the *SOX2−* populations in HN120PCR. Surprisingly, in vivo LDA revealed that both *SOX2*+ and *SOX2−* (from HN120PCR) populations displayed equivalent tumor-initiating potential (Fig. 4f). These observations invoked the possibility of other stem factors that may be involved in fueling the growth and proliferation of *SOX2−* tumors (Fig. 4g).

### Drug-induced lineage infidelity leads to adaptive evolution

Our results thus far indicated that the loss of bona fide *SOX2*+ CSCs did not impact the tumor-initiating potential of HN120PCR cells, thereby suggesting a potential switch in stem cell hierarchy. However, this does not rule out the possibility of another stem cell pool (distinct from SOX2+) in HN120Pri cells that may lead to drug-induced HN120PCR state. We investigated this by applying RaceID2 algorithm^35^, which allows the identification of cell-of-origin from scRNA-seq data. We hypothesized that if there is common cell-of-origin for both HN120Pri and HN120PCR then RaceID2 will be able to identify this cell type. Interestingly, although RaceID2 indicated increased stem-potential of HN120PCR cells, it could not identify a common ‘cell-of-origin’ between HN120Pri and HN120PCR (Fig. 4h). Importantly, RaceID2 faithfully identified the common origin of HN137Pri and HN137 PCR (Fig. 4i), providing further evidence for clonal selection of CSCs in the HN137 model while drug-induced lineage infidelity of CSCs in HN120 model. The lack of a common lineage between HN120Pri and HN120PCR cells was further validated by Psuedo-temporal ordering of these cells by Monocle2.0^36^. Psuedo-temporal ordering of HN120 populations revealed discontinuity between the parental and the metastatic/drug-resistant lineages, thereby suggesting a stress-induced cell-state switch (Supplementary Figure 8e). On the contrary, for HN137Pri we observed a continuum that branched out into two separate drug-resistant (HN137PCR) and metastatic (HN137Met) lineages, corroborating the clonal selection hypothesis (Supplementary Figure 8f). Both methodologies strongly implicated the retention of an original stem cell pool in HN137 model, and a switch in stem cell hierarchy in HN120, under the selection pressure of drug treatment or metastasis.

### Interplay of *SOX2* and *SOX9* modulates the stress-induced stem cell switch

**Fig. 5:**
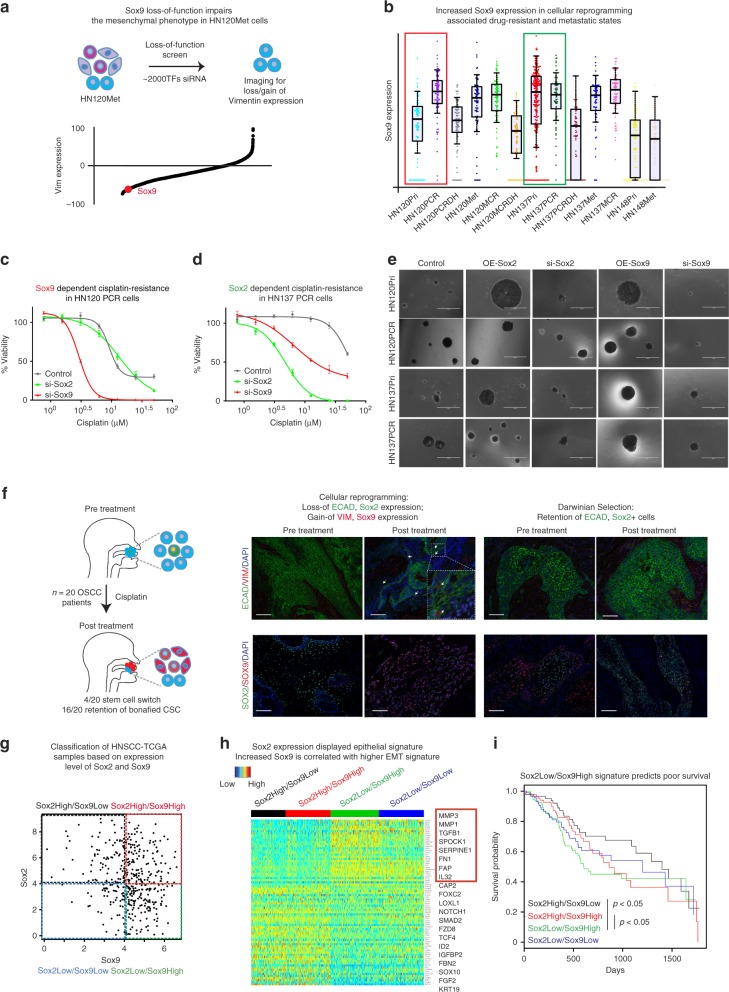
Pioneer factor SOX9 drives drug-induced cellular reprogramming and marks the acquired mesenchymal cell state. **a** Identification of the master regulators of mesenchymal cell fate, as analyzed by Vimentin expression, by an siRNA screen for ~2000 transcription factors in HN120Met. SOX9 was identified as a top candidate hit, the knockdown of which resulted in a marked loss of Vimentin expression. **b** Beeswarm plot of SOX9 expression in different cell types from 1302 scRNA-seq libraries. **c**, **d** Dependency of SOX2 and SOX9 on cisplatin resistance in HN120PCR/HN137PCR models. Graphs depict dose-dependent viability of resistant models treated with cisplatin. Loss-of SOX9 but not SOX2 reversed cisplatin-resistance in HN120PCR (**c**), while siRNA-mediated knockdown of SOX2, but not SOX9 conferred a significant loss of cisplatin-resistance in HN137PCR cells (*n* = 3, mean + s.e.m. of biological and technical replicates). **e** Sphere-forming assays with 500 single cells from HN120Pri, HN120PCR, HN137Pri, and HN137PCR cells. Shown are the effect of genetic loss/gain-of-function of SOX2 and SOX9 on sphere forming ability of treatment naïve and resistant cells (*n* = 3). **f** Immunofluorescence-based expression analysis of lineage markers (ECAD in green and Vimentin in red) and stem cell factors (SOX2 in green and SOX9 in red) in FFPE-section of matched OSCC patient tumors: treatment naive and locally recurrent. Inset demonstrating the de novo emergence of Vimentin expressing cells; note the SOX2 to SOX9 switch in these tumors. **g** Virtual sorting of TCGA-HNSCC in SOX2^High^/SOX9^Low^, SOX2^High^/SOX9^High^, SOX2^Low^/SOX9^High^, and SOX2^Low^/SOX9^Low^ sub-sets. **h** Differential expression of EMT markers (in red rectangle) in sub-sets based on the “sorted” SOX2 and SOX9 populations. **i** Five-year survival analysis of the SOX2/SOX9 subsets identified in (**g** and **h**). Note patient with SOX2^Low^/SOX9^High^ signatures display poorer survival (Cox regression *P* value < 0.05). Scale bar = 1000 μM (**e**), 50 μM (**f**)

The loss of *SOX2*, retention of tumor-initiating capabilities, and the ‘switch-like’ behavior observed in HN120 drug-resistant and metastatic counterparts suggested the possibility for a stem cell switch. In search for a putative master regulator that could affect this switch, we conducted an RNAi-screen for transcription factors to identify modulators of the mesenchymal fate in HN120Met. Remarkably, amongst the top hits we recovered *SOX9* (Fig. 5a), a key stem factor reported to be co-expressed in *SOX2+* tumor-initiating SCCs^37,38^. Moreover, *SOX9* has recently been demonstrated as a pioneer factor for stem cell plasticity during wound healing and cancer^32,39^. Therefore, we investigated whether *SOX9* could function in cisplatin-/metastasis-induced cellular reprogramming of HN120Pri cells. We observed that the loss of *SOX2*+ cells in HN120PCR was associated with a concomitant gain in *SOX9* expression (Fig. 5b and Supplementary Figure 10a). Curiously, we observed anti-correlation between *SOX2* and *Vimentin* expression, thereby suggesting that *SOX2* is required for maintenance/retention of the epithelial phenotype. In contrast, the vast majority of *SOX9*+/*SOX2−* cells displayed robust expression of vimentin (Supplementary Figure 10b). We further validated these observations by immunofluorescence and confirmed that *SOX9* and *Vimentin* are indeed co-expressed in *SOX2−* cisplatin-resistant (HN120PCR) and metastatic HN120 cells (Supplementary Figure 10c). To determine the effect of *SOX2* and *SOX9* levels on stem cell-state, epithelial or mesenchymal cell fates in OSCC we performed siRNA mediated loss of function experiments. We observed that loss of *SOX2* reduced the expression of epithelial markers while increasing the expression of mesenchymal genes in HN120 and HN137 models (Supplementary Figure 11a-d). In contrast, the loss of *SOX9* lead to an increase in epithelial gene expression with a concomitant reduction of mesenchymal properties (Supplementary Figure 11a-d and Supplementary Table 2). Altogether, these results suggest a possible role for the interplay between *SOX2* and *SOX9* in maintenance of epithelial and mesenchymal cell-states in OSCC.

Next, we sought to investigate the functional implication of *SOX2* and *SOX9* in maintenance of drug-resistant properties. siRNA-mediated knockdown of *SOX9* but not *SOX2* markedly enhanced the cisplatin-mediated killing of HN120PCR cells, confirming the requirement for *SOX9* as a driver of resistance in *SOX2*-negative cells (Fig. 5c). Conversely, HN137PCR cells demonstrated strong dependence on *SOX2* expression to retain cisplatin-resistance (Fig. 5d). To test the implication of *SOX2* and *SOX9* in maintenance of stem phenotype, we performed sphere forming assays with cells displaying gain/loss-of *SOX2* or *SOX9* expression. HN120Pri, HN137Pri, and HN137PCR cells clearly indicated a positive correlation between levels of *SOX2* expression and their sphere forming ability. Remarkably, the HN120PCR cells switched their dependency from *SOX2* to *SOX9* (Fig. 5e). Notably the loss either *SOX2* or *SOX9* in HN137PCR and HN120PCR respectively, resulted in reduced expression of the putative stem marker *ALDH*, further corroborating the notion of alternative dependencies on stem factors between the two models (Supplementary Figure 10d). Taken together, these results validate the divergent role of *SOX2* and *SOX9* in maintenance of drug-resistance and cell-state phenotypes in OSCC.

### Validation of drug-induced stem cell plasticity in OSCC patients

To validate whether the interplay between *SOX2* and *SOX9* stem cell program is broadly applicable to OSCC patients undergoing cisplatin-treatment, we extended our observation to four additional patient-derived models along with examining the expression of stem (*SOX2/SOX9*) and lineage (*ECAD/VIM*) markers in a larger patient cohort (*n* = 20). First in cell line models, we observed cisplatin-resistance can be achieved either by ‘selection’ of epithelial (SOX2+, ECAD+) cells or ‘reprogramming’ into mesenchymal (SOX9+, VIM+) states. Cisplatin treatment resulted in elevated expression of Vimentin and SOX9 in PDPC models HN-26 and HN-43 (reprogramming), while HN-19 and HN-64 demonstrated gain of SOX2 and ECAD levels, suggesting clonal evolution (Supplementary Figure 12). These results further corroborated and extended the observations supporting the divergent modes of drug-resistance observed in HN120 and HN137 PDPC models.

Next to validate the drug-induced stem cell switch in ‘microenvironment competent’ physiological conditions in the clinic we examined the expression of *SOX2* and *SOX9* in prospective cohorts of matched SCC tissues obtained from treatment naive and locally recurrent tumors from OSCC patients (*n* = 20). In concordance with data obtained from patient-derived cells line models, drug-induced cellular-reprograming (de novo emergence of Vimentin) was associated with the concomitant gain of SOX9 and the loss of SOX2 expression (*n* = 4/20) (Fig. 5f), whereas the fixation/selection of the epithelial cell-states was associated with retention of SOX2 expression (*n* = 16/20). These results demonstrated the reproducibility of drug-induced stem cell switch and the consequent cellular-reprograming in the SCC patient tumors, thereby underscoring the function of cell-intrinsic properties in modulating resistance. Further clinical validation were performed on the expression analyses of TCGA datasets from HNSCC and lung SCCs. Based on the expression of *SOX2* and *SOX9*, we sub-divided the HNSCC-TCGA data into four cohorts: *SOX2*^*High*^*/SOX9*^*Low*^, *SOX2*^*High*^*/SOX9*^*High*^, *SOX2*^*Low*^*/SOX9*^*High*^, and *SOX2*^*Low*^*/SOX9*^*Low*^ (Fig. 5g). Interestingly, both *SOX2*^*High*^ groups irrespective of *SOX9* status demonstrated lower expression of mesenchymal markers, revealing a correlation between *SOX2* expression and the epithelial cell state (Fig. 5h). On the contrary, the *SOX9*^*High*^ cohort displayed elevated mesenchymal markers, corroborating the notion that the loss-of *SOX2* and gain-of *SOX9* promotes a mesenchymal cell-state. Notably, the HNSCC patient cohort represented by *SOX2*^*Low*^*/SOX9*^*High*^ demonstrated poorer 5-year overall survival compared to the *SOX2*^*High*^*/SOX9*^*Low*^ patients (Fig. 5i; *p* < 0.05), suggesting that the former represents the more aggressive phenotype. We observed similar trends in lung SCC where patients with high *SOX2* expression demonstrate an epithelial-like gene-signature, while *SOX9*^*High*^ expression was associated with mesenchymal features (Supplementary Figure 13a-c and Supplementary Table 3). Altogether these observations suggested a critical interplay between *SOX2* and *SOX9* in promoting a phenotypic switch during the acquisition of mesenchymal-like states, the maintenance of stem cell identity, and tumor-initiating properties in SCC.

### Poised epigenetic state facilitates *SOX9*-mediated cellular reprograming

**Fig. 6:**
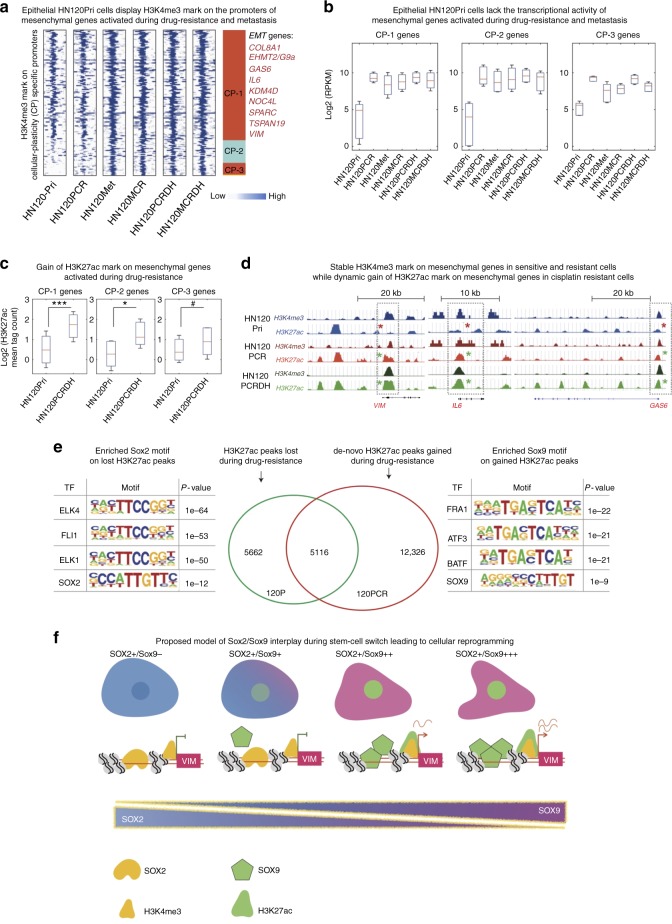
Poised mesenchymal promoters in treatment naive epithelial cells. **a** Chip-seq analysis for H3K4me3 in HN120 naïve, resistant and drug-holiday models. H3K4me3 marks on promoters of cisplatin-induced genes associated with cellular-reprogramming (EMT) in HN120 cells. Clusters CP-1 and CP-2 represent poised promoters, whereas CP-3 is associated with closed promoters. **b** Median expression of cisplatin-dependent upregulated genes from HN120 cells in clusters 1–3 (from Fig. 6a). **c** Quantitation of H3K27ac signals on promoters of cisplatin-dependent up-regulated genes in clusters 1–3 (from Fig. 6a) **b**, **c** mean + s.d., Mann–Whitney *U* test, ***P < 0.0005, *P < 0.005, #non-significant). **d** ChIP-seq tracks of H3K4me3 and H3K27ac on *VIM*, *IL6,* and *GAS6* promoters in HN120Pri naive and drug-resistant/holiday models. Note the gain of H3K27ac marks in PCR/PCRDH cells (green asterisks), compared to naive (red asterisks), on EMT-associated promoters (*y*-axis = 0–75 (*VIM*, *GAS6*), 0–25 (*IL6*)). **e** Venn-diagram representing differential H3K27ac peaks in drug-sensitive and drug-resistant HN120 cells. HOMER based discovery of de novo transcription factor motifs in differential H3K27ac peaks. Note the loss of SOX2 motif in the drug-resistant HN120PCR cells, and the enrichment for SOX9 binding sites in the de novo acetylated promoters/enhancers. **f** Proposed model for SOX2 and SOX9 interplay during stem cell switch. Our data suggests that SOX2 expression maintains the epithelia cell-state while the loss of SOX2 and a concomitant gain of SOX9 results in the activation of an EMT program in HN120 drug-resistant and metastatic tumor cells

The data thus far demonstrated that cisplatin and metastatic-selection induced remarkable phenotypic plasticity in HN120Pri cells by switching stem cell states. Recent reports have implicated the function of chromatin remodeling in regulating cell-state transitions^11,40^. More importantly, *SOX9* has been shown to modulate the chromatin dynamics and cellular-plasticity in skin cancers^39^. Therefore, we decided to investigate whether cisplatin and metastasis-mediated cell-state transition in HN120 cells could be the result of epigenetic rewiring. We performed ChIP-seq to examine the chromatin status of mesenchymal genes in cisplatin-resistant and metastasis cells (Supplementary Data 5). First, we assessed the H3K4me3 mark on the promoters of differentially upregulated genes in HN120PCR compared to HN120Pri cells. As shown in Fig. 6a, promoters of mesenchymal-associated genes were already marked with the H3K4me3 in treatment naive HN120Pri cells, despite not being expressed (Fig. 6b). We speculate that the lack of expression could be due to the presence of bivalent histone marks on poised promoters. Chip-PCR of Vim promoter in the HN120Pri revealed that indeed it was decorated both with H3K4me3 as well as H3K27me3, which is associated with repression of transcription (Supplementary Figure 14a). We therefore reasoned that these promoters existed in a poised-state and additional epigenetic modulation lead to cellular-reprogramming during metastasis and drug-resistance. To investigate this, we performed H3K27ac ChIP-seq to identify active chromatin marks in HN120 primary, metastatic, their drug-resistant and drug-holiday cells. The poised promoters of EMT-associated genes demonstrated a marked gain of H3K27 acetylation, correlating with their activation status (Fig. 6c and Supplementary Figure 14b). Specifically, key EMT-associated genes (*VIM*, *IL6,* and *GAS6*) were already decorated with H3K4me3 in naive primary cells and subsequently gained acetylation during metastasis and drug-resistance (Fig. 6d). Notably, we also observed a concomitant loss of the H3K27me3 repressive mark on the VIM promoter suggesting that the expression of plasticity-associated genes is tightly regulated by the factors modulating epigenetic rewiring (Supplementary Figure 14). Next, we employed HOMER (Hypergeometric Optimization of Motif EnRichment) to identify de novo transcription factor motifs enriched in differential H3K27ac peaks in HN120Pri and HN120PCR cells. Intriguingly, we found enrichment of SOX2 motifs in the HN120Pri-specific peaks, while the peaks gained by drug-resistant HN120PCR cells demonstrated an overrepresentation of the SOX9 motif, in addition to other stress-induced transcription factors (FRA1, ATF3, BATF) (Fig. 6e). These results are in concordance with expression and siRNA validation data where HN120Met/HN120PCR cells demonstrated higher expression and dependency on *SOX9* levels. Altogether, these data suggest that the epithelial HN120 cells harbor a poised mesenchymal chromatin. Cisplatin treatment or metastatic-selection provides a ‘stress signal’ resulting in the elevation of SOX9 expression that drives cellular-reprogramming by inducing H3K27 acetylation, and thus transcriptional activation of poised ‘EMT’ promoters, leading to trans-differentiation.

### *BRD4* inhibition reverses cisplatin-resistance in SCC cells

**Fig. 7:**
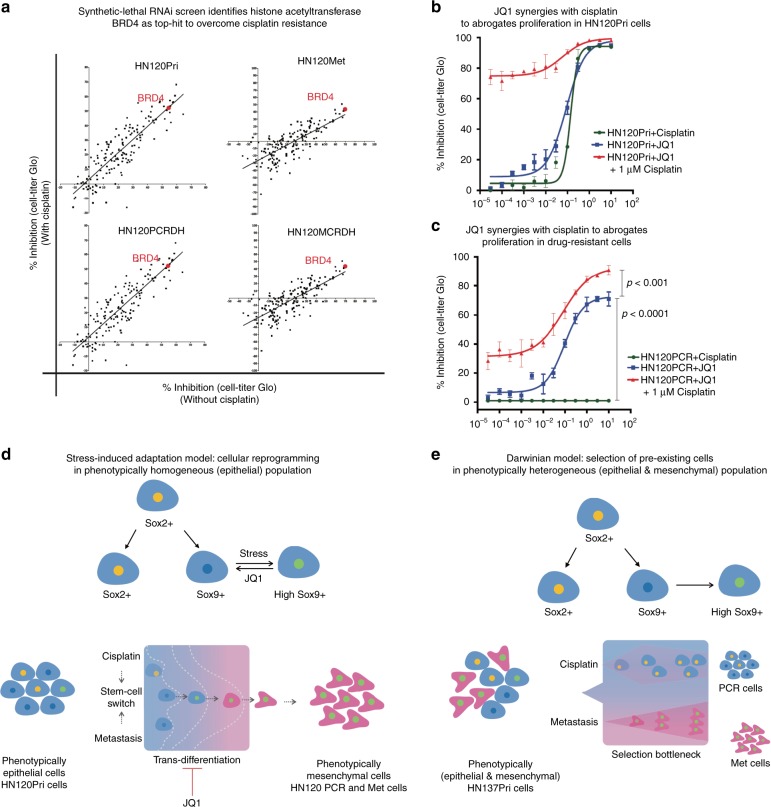
Different modes of drug-resistance uncover potential for combinatorial targeting strategies using epigenetic inhibitors. **a** Synthetic-lethal screens with cisplatin using siRNAs against chromatin remodelers (see Fig. S6 for complementary screen using small molecule inhibitors). IC50 curves for cell viability screens in naive (**b**) and drug-resistant (**c**) HN120 cells with increasing concentration of either cisplatin or JQ1 alone, or a combination of JQ1 with 1 µM cisplatin (mean + s.e.m. *n* = 3, Two-tail Student’s *t* test, ***P < 0.0001, *P < 0.001). **d**, **e** Proposed model for cellular-reprogramming (adaptive) and ‘clonal selection’ modes of drug-resistance and metastasis. **d** The phenotypic homogeneous (HN120) model depicts the adaptive mode of evolution where epigenetically poised epithelial cell populations undergo mesenchymal cell-state transition. Different stress (cisplatin or metastasis) conditions leads to similar phenotypic outcome as a result of a SOX9-mediated coordinated activation of H3K4me3- poised promoters, which can be reversed by JQ1 mediated BRD4 inhibition. **e** Tumor cells exhibiting pre-existing phenotypic heterogeneity (HN137) allows “division-of-labor” where distinct clonal populations of pre-existing ‘designated survivors’ can get selected depending on the context specific nature of different selection pressures

Since, stress-induced trans-differentiation was associated with a camouflaged epigenetic state, we screened for CRs, and epigenetic drugs/small molecule compounds that may modulate this process. Comprehensive synthetic lethal screens with siRNAs (*n* = 154) and small molecule epigenetic drugs (*n* = 42) identified both *BRD4* (a histone acetyltransferase) (Fig. 7a) and JQ1 (a potent inhibitor of BRD4) (Supplementary Figure 15)^41^. Further, we investigated whether JQ1 treatment can suppress acetylation and activation of EMT-associated genes. Indeed, short-term JQ1 treatment (12 h) diminished H3K27 acetylation, and subsequent gene expression from the Vimentin promoter in HN120-PCRDH cells (Supplementary Figure 16). Therefore, we hypothesized that JQ1 treatment could potentially inhibit the observed cellular-plasticity, and therefore re-sensitize the drug-resistant cells to cisplatin-treatment. Indeed, JQ1 treatment induced the cisplatin sensitivity in both naive (HN120Pri) and resistant HN120PCR cells, suggesting that combinatorial treatment with JQ1 and cytotoxic drugs could potentially reverse and/or delay the onset of resistance (Fig. 7b, c). These results underscore the importance of epigenetic modulation as a therapeutic strategy to overcome adaptive mechanisms that result in the evolution of metastatic dissemination and resistance against cytotoxic drugs.

## Discussion

Identifying the extent of ITH and its impact on tumorigenesis, drug-resistance, and metastatic dissemination remains a major focus in the field of cancer research. Amongst the variety of methodologies available to identify genetic and phenotypic heterogeneity, single cell transcriptomics has more recently emerged as a powerful and revolutionary approach for in depth exploration of ITH. To understand the impact of ITH on evolutionary trajectories of cancer cells it is important to generate reliable and clinically relevant model systems. Therefore, we employed paired synchronous primary and metastatic PDPC models^21^ to evaluate the impact of drug treatment in these populations, and also investigate the evolutionary trajectories of the drug-induced, vs. metastatic states.

The commonly held view is that higher degree of diversity at the population level could provide selective advantage during drug-treatment and/or metastasis. Broadly, epithelial tumors exhibit two major cell types, epithelial and mesenchymal. Among these the latter is frequently associated with poor prognosis and drug-resistance. In our experimental system, we chose two PDPCs, one displaying an epithelial phenotype (HN120Pri) and the other a mixture of epithelial and mesenchymal sub-populations (HN137Pri). Surprisingly, under cisplatin-induced or metastatic selection, we observed the de novo emergence of the mesenchymal cell state from the otherwise homogeneous primary epithelial cells (HN120Pri). The inherent plasticity exhibited in the HN120Pri populations represents a moving therapeutic target because it can remain ‘hidden in plain sight’, but dynamically switch phenotypes upon the application of selection pressure (Fig. 7d). On the other hand, in HN137Pri displayed classical ITH where various phenotypic sub-populations pre-existed in the parental cells, and the nature of differential selection pressures enriched for either epithelial or mesenchymal states. This is presumptively a consequence of ‘division-of-labor’ where specialized sub-populations display characteristics that enable their selection as ‘designated survivors’. ‘Division-of-labor’ depicts the prototypical form of ITH that can be easily documented, characterized, and potentially targeted using combinatorial strategies (Fig. 7e).

Phenotype switching can allow cells to acquire diversity under selection pressure of unfavorable conditions. HN120 cells displayed cellular-reprogramming where under stressful conditions the epithelial cells consistently adopt a mesenchymal fate. Most strikingly, the paired metastatic (HN120Met) model, originally isolated from the lymph node metastatic site of patient-HN120, also displayed a remarkable similarity in transcriptome and phenotype, when compared to HN120PCR. These observations emphasize the utility of synchronous patient-derived models, while also indicating a molecularly defined, deterministic mechanism for the observed phenotypic switch. We noted that the phenotypic switch was associated with a loss of *SOX2* expression, an important determinant of tumorigenic properties in SCCs. Surprisingly, loss of *SOX2* expressing cells did not result in reduced tumorigenic potential as the population adapted to a *SOX9* dependent stem cell state. This was validated by the lineage-infidelity observed in RaceID2 and monocle as well as the dependence of drug-resistant HN120PCR cells on *SOX9* expression.

It important to note that *SOX9* has been recently linked to wound healing and lineage-infidelity, in SCCs^32^. Our data suggests that *SOX9* may serve as a molecular rheostat that can ‘sense’ the stress in cellular microenvironment. Both metastasis and cisplatin-induced selection led to a gain in SOX9 expression, which resulted in the switch from a *SOX2* to *SOX9* dependent stem cell state in HN120Pri. Importantly, both *SOX2* and *SOX9* have been shown to play important functions in maintenance of stem cells and tumorigenesis. However, *SOX2* is known to specify cell-fates by antagonizing other transcription factors^42^. Indeed, we made similar observations in our models where the *SOX2*+/*SOX9*+ populations primarily exhibited epithelial characteristics, while the loss of *SOX2* expression resulted in the transition to a mesenchymal fate. This was also observed in cell line models where loss of *SOX2* expression led to increased mesenchymal properties. These results suggest that presence of *SOX2* may antagonize the *SOX9* associated transcriptional program. Our studies allowed us to identify distinct stem cell states in SCC and their role in cell-fate decisions. Importantly, these distinct stem-states were identified in larger cohorts of pre-cisplatin and post-cisplatin treated HNSCC patients as well as in TCGA dataset, where higher *SOX9* expression correlated with elevated mesenchymal properties and poor survival, compared to *SOX2* expression. Taken together, our data provides new insights into a possible threshold-dependent interplay between these two pioneer factors.

Cellular-reprogramming has been associated with chromatin state of the cells. We speculated that phenotype switching is associated with gain of de novo chromatin sites specifically at EMT associated/driver genes. On the contrary we observed promoters of EMT genes were already marked by H3K4me3 in HN120Pri cells suggesting poised state of mesenchymal promoters in epithelial cells. Interestingly, poised chromatin may provide a landscape for covert cell-states where epigenetically mesenchymal cells can be camouflaged as phenotypically epithelial. Furthermore, we identified *BRD4* as a critical histone acetyltransferase involved in the process of cell fate switching by promoting H3K27-acetylation on EMT promoter/enhancers. JQ1 mediated short-term inhibition of *BRD4* reversed the cisplatin-resistance in HN120PCR and HN120Met cells. It is important to note that *SOX9* has been linked with H3K27ac of super-enhancers in cells displaying phenotypic plasticity. Our data suggests that *SOX9* in combination with *BRD4* (and other CRs) may result in the opening of poised chromatin. Future research is warranted to identify the critical threshold of *SOX9* that is required to maintain ‘passive’ vs. ‘induced’ status of EMT promoters and enhancers^14^.

While single cell approaches have been extensively used to identify and characterize novel cell types and dissect intra-tumor/inter-patient heterogeneity, there have limited efforts on investigating tumor evolution using longitudinal, isogenic patient-derived/PDPC models. This is important not only because PDPCs can be used for *in plate* modelling of tumor evolution under different selection pressures (drugs, hypoxia, metastasis), they also provide an excellent platform for functional validation for in-depth characterization of the molecular mechanisms driving evolutionary trajectories. Importantly, insights gained from such studies can be cross-validated in samples from retrospective or prospective patient cohorts in the clinic to accelerate the process of clinical translation. As is amply evident from our study, we could robustly validate the phenomenon(s) of drug-induced emergence of de novo cell state, and stem cell-switch observed in our in vitro PDPC models in matched patients, as well as prospective patient cohorts under the setting of a neo-adjuvant clinical trial. Additionally, the revelation of the *SOX2* to *SOX9* stem cell switch allowed us to identify a new molecular subtype in the TCGA cohorts for SCCs, namely *SOX2*^*lo*^*; SOX9*^*hi*^, which is prognostic for survival. It is therefore tempting to speculate that in the future, clinical implementation of epigenetic inhibitors in combination with metronomic dosing schedules can prevent cells from activating stress-response mechanisms that may switch their dependency to alternative stem factors, and hence their survival. Altogether our results underscore the remarkable predictive power of PDPC and scRNA-seq technology in predicting not only the course of tumor evolution in the clinic, but also in revealing novel mechanistic insights that can be harnessed to design the next generation of ‘evolutionary therapeutic’ strategies.

## Methods

### Generation and passaging of PDXs

Tumor samples were obtained from OSCC patients post-surgery after obtaining informed patient consent in accordance to SingHealth Centralized Institutional Review Board (CIRB: 2014/2093/B). Tumors were minced into ~1 mm^3^ fragments and suspended in a mixture of 20% Matrigel (Corning, cat. no. 354234) in DMEM/F12 (Thermo Fisher, cat. no. 10565–018). The tumor fragment mixtures were then implanted subcutaneously into the left and right flanks of 5–7 weeks old NSG (NOD.Cg-*Prkdc*^*scid*^ *Il2rg*^*tm1Wjl*^*/*SzJ) (Jackson Laboratory, stock no. 005557) mice, using 18-gauge needles. Tumors were excised and passaged when they reached 1.5 cm^3^. For passaging, tissues were cut into small fragments of 1 mm^3^ prior to resuspension in 20% Matrigel/DMEM/F12 mix, before subcutaneous inoculation of tumor fragments into 5–7 weeks old NSG mice.

### Derivation of PDPC cell lines and cell culture

Tumors were minced prior to enzymatic dissociation using 4 mg/ml Collagenase type IV (Thermo Fisher, cat. no. 17104019) in DMEM/F12, at 37 °C for 2 h. Cells were washed using cyclical treatment of pelleting and resuspension in phosphate buffered saline (PBS) (Thermo Fisher, cat. no 14190235) for three cycles. The final cell suspensions were strained through 70 µm cell strainers (Falcon, cat. no. 352350), prior to pelleting and resuspension in RPMI (Thermo Fisher, cat. no 61870036), supplemented with 10% fetal bovine serum (Biowest, cat. no S181B), and 1% penicillin-streptomycin (Thermo Fisher, cat. no. 15140122). Cells were kept in a humidified atmosphere of 5% CO_2_ at 37 °C. Cell line identity was authenticated by comparing the short tandem repeat (STR) profile (Index BioResearch) of each cell line to its original tumor^21^.

### Generation of PDX cisplatin resistant model

The HN120Pri (0.25 million) and HN137Pri (1,000,000) cells were trypsinzed to dissociated into single cell suspension followed by subcutaneous implantation into the left and right flanks of 5–7 weeks old NSG (NOD.Cg-*Prkdc*^*scid*^ *Il2rg*^*tm1Wjl*^*/*SzJ) (Jackson Laboratory, stock no. 005557) mice, using 18-gauge needles. Tumor growth was followed every three days and after 2–3 weeks of growth, when tumor volume reaches ~ 100 mm^3^, animals were divided into control and treatment cohorts. The treatment group was treated with 3 mg/kg body weight of cisplatin for every three days and control group received DMSO as vehicle control. Three week post-treated tumors were harvested from cisplatin and control groups for dissociation into single cell suspension followed by flow-cytometric analysis of cell-type and stem-state markers. Flow-cytometery data was analysed by FlowJo.

### Immunofluorescence assays

For immunofluorescence, paraformaldehyde-fixed, paraffin-embedded tissue sections were first deparaffinised and rehydrated. Heat mediated antigen retrieval was then performed in a pressure cooker at 120 °C for 20 min in Tris/EDTA buffer (pH9, DAKO, S2367). Tissue sections were incubated in primary antibody overnight at 4 °C. The primary antibodies used were: rabbit anti-Vimentin (1:100, Abcam, ab16700), mouse anti-E-cadherin (1:50, Abcam, ab76055), rabbit anti-Sox9 (1:200, Abcam, ab185966) and mouse anti-Sox2 (1:200, Cell Signaling Technology, L1D6A2). Tissue sections were subsequently incubated in secondary antibodies for 1 h at room temperature before mounting in Hydromount (EMS, 17966) with Hoescht 33342 (1:1000, Thermo Fisher Scientific, H3570). The secondary antibodies used were anti-chicken/rabbit/mouse conjugated to Alexa 488/647 IgG (1:500, Life Technologies). Immunostained slides were imaged with Vectra 3.0 Automated Quantitative Pathology Imaging System and image analysis was conducted using inform Cell Analysis software (Perkin Elmer). For immunocytochemistry assays primary cells were fixed with aceto-methanol followed by staining with anti-Vimentin (1:200, Abcam, ab16700), mouse anti-E-cadherin (1:200, Abcam, ab76055), rabbit anti-Sox9 (1:200, Abcam, ab185966) and mouse anti-Sox2 (1:200, Cell Signaling Technology, L1D6A2), anti-Cytokeratin 5 antibody (1:200, Abcam, ab52635) and anti-Cytokeratin 14 antibody (1:200, Abcam, ab181595) antibodies and imaged on PerkinElmer Operetta High-Content Imaging System. EdU pulse chase assay was performed using Click-IT^TM^ EdU Alexa Fluor^TM^ 555 Imaging Kit (Catalog Number C10338).

### Flow cytometry

Tumor were harvested from control or treated mice, and digested with collagenase IV (Life Technologies), passed through a 70 µm followed by 40 µm cell strainers (BD Falcon) and washed with PBS. Red blood cells (RBCs) were lysed using RBL (RBC Lysing Buffer, Bio Legend). Single cells suspensions were labelled according to standard procedures. Cells were stained with rabbit anti-Vimentin (1:200, Abcam, ab16700), mouse anti-E-cadherin (1:200, Abcam, ab76055), rabbit anti-Sox9 (1:200, Abcam, ab185966) and mouse anti-Sox2 (1:200, Cell Signaling Technology, L1D6A2).

The gating strategy for viable singlets is shown in Supplementary Figure 3a.

### Single cell RNA-seq

The C1 Single-Cell Auto Prep IFC (Fluidigm) system was employed for single-cell RNA-seq. Single cells were captured using medium and/or large size chips, followed by reverse transcription and cDNA pre-amplification in the C1-Chips using the SMARTer PCR cDNA Synthesis kit (Clontech) and the Advantage 2 PCR kit (Clontech). The single-cell libraries were prepared using the Nextera DNA Sample Preparation Kit and the Nextera Index Kit (Illumina).

### mRNA amplification, library construction, sequencing, and processing

PDPC single-cell were prepared by Fluidigm-C1 system. Briefly, PDPC were cultured in RPMI-1640 with 10% serum and antibiotic. All the drug-resistant lines were constantly maintained in cisplatin (respective final dose for each cell line- Supplementary Table 1). Single cell suspension was achieved by trypsinisation followed by loading in C1 microfluidic 96-well plates (medium size) as per manufactures guidelines. The mRNA and cDNA was harvested in C1 system followed by barcoding in 96 well plate. We employed Nextra XT Library Prep kit (Illumina) for library tegmentation. The tegmented single-cell libraries were amplified by 12–14 round of PCR cycle. The libraries from 96 cells with unique barcoded were pooled and sequenced using a HiSeq-Hi-output-2500 sequencer (Illumina).

### Sequencing data pre-processing for single-cell RNA-seq

The raw reads in FASTQ files were aligned to the human genome (hg19 assembly) using Tophat-2.1.0^43^. The BAM files thus obtained were then used by Cuffdiff-2.2.1^44^ for quantifying expression. The read counts were divided by the length of each gene to obtained reads per kilobases (RPK). These RPK values were divided by ‘per million’ scaling factor to obtain transcript per kilobase million (TPM). Expression of the RefSeq genes were then used for downstream bioinformatics analysis.

### ChIP-seq

For chromatin preparation one million cell were cross-linked (10% FBS/DMEM containing 1.5% PFA) for 30 min. Followed by addition of 1/20 volume of 2.5 M glycine, next these cells were incubated on ice for 5 min. Crosslinked cellular material was re-dissolved in buffer containing 10 mM Tris-HCl, pH8, 1 mM EDTA, 0.5 mM EGTA, 1X protease inhibitor cocktail (Roche) followed by sonication for 10–12 cycles (30 s on/off). Chromatin was pulled by incubation with H3K4me3 or H3K427ac antibody-coupled Dynabeads and 150 μl of ChIP buffer (433 mM NaCl, 0.43% sodium deoxycholate, 4.3% Triton X-100). The 1/10 of chromatin mixture was separated for input control. The beads were washed 5× with RIPA buffer and incubated in 100 μl of elution buffer (50 mM Tris-HCl, pH8, 1% SDS, 10 mM EDTA) at 65 °C for 20 min with brief vortexing followed by recovery of immunoprecipitated chromatin. The immunoprecipitated chromatin and input samples were de-crosslinked by incubation at 65 °C overnight and subsequent incubation with 0.2 μg/μl of RNase A at 37 °C for 1 h and with 0.2 μg/μl of proteinase K at 55 °C for 2 h. The DNA was extracted by phenol/chloroform/isoamylalcohol (49:49:2) and ethanol precipitated in presence of 40 μg of glycogen. After washing with 70% ethanol, pellets were resuspended in 10 mM Tris-HCl, pH8. Multiplexed ChIP-seq libraries were generated using NEBNext ChIP-seq library Prep kit (NEB) following the instructions from the manufacturer. The sequencing was performed on Illumina HiSeq-2500. We employed D-filter^45^ to call ChIP peaks in the naive, drug-resistant and drug-holiday H3K4me3 and H3K27ac ChIP-seq libraries relative to input controls.

### Synthetic lethal screens

We employed high throughput siRNA screens (HTS) to investigate the functional role of CRs in cisplatin-induced cellular plasticity. To probe the role of chromatin in drug-resistance we individually transfected 154 siRNA associated with chromatin remodeling in naive and drug-resistant HN120 cells. Followed by incubation of these cells with cisplatin for 72 h and evaluation of cell proliferation. This allowed us to identify CRs involved in cisplatin-induced drug-resistance. Based on this screen we performed associated screen with small molecule inhibitors (SMIs) of chromatin. The OSCC PDPC were cultured with 42 SMIs in the presence and absence of cisplatin for 72 h followed by evaluation of cell proliferation. To identify the lineage determining factor, we individually transfected ~2000 siRNA associated with transcription factors in *SOX2*-negative HN120Met cells. This was followed by imaging of these cells for Vimentin expression post 72 h of transfection. This allowed us to identify stem cell factor involved in cellular-reprogramming.

### Clustering of single-cell libraries based on RaceID analysis

To identify rare cell population of drug-resistant cells we employed RaceID as described by Grün et al.^26^. Based on quality control parameter, a total of 1302 single-cell libraries were used for analysis. Briefly we normalized the data by dividing transcript counts in each cell by the total number of transcripts in these cells followed by multiplication with the median (m) of the total number of transcripts across the cells. We further filtered the low expressed genes by discarding genes which are not expressed at one (minexpr) transcript in at least five (minnumber) of cells. The RaceID provided five clusters which were further projected on t-SNE map to reduce the dimensionality of complex single-cell libraries.

### Differential gene expression

To identify cluster specific genes, we calculated the median expression across all cells in each cluster. Next, we determined the fold-change of each gene in given cluster vs. rest of the clusters. The top-differential genes were determined based on *p*-value calculated by binomial counting statistics. Fold-change was determined by the median expression difference between given cluster vs. rest of the cells. We focused on top-50 most-differentially up and down-regulated genes (total 100) from each scRNA-seq cluster.

### Principle component analysis

Principal Components Analysis (PCA) was performed on quantile-normalized TPM followed by log2 transformation with pseudo-counts of 1 added. Pre-ranked GSEA (http://software.broadinstitute.org/cancer/software/genepattern/modules/docs/GSEAPreranked/1) for hallmark and Gene Ontology (GO) gene sets was applied to the set of 2000 genes with highest absolute loadings for each of the principal components. We used the principal component score vectors of the most informative PC1 and PC2 to determine the Euclidean distance between cells. Hierarchical clustering was then performed on the distance matrix.

### Pathway and gene set overdispersion analysis

We applied PAGODA to investigate the variance explained by the first and second principal component of Hallmark and GO gene sets (http://software.broadinstitute.org/gsea/msigdb). The algorithm allows identification of the most over-dispersed gene sets. In order to reduce redundancy, gene sets showing similar expression patterns were integrated into aspects using a distance threshold of 0.5. Subsequently, cells were clustered based on a weighted correlation of genes that drive the significant aspects and the heatmap highlight the most variable aspects. Correlation from the hierarchical clustering were used to visualize cells in two dimensions using t-SNE plots. Cell states or types following Pagoda cluster identification were assigned using the most variable genes. Pagoda defines these genes using a weighted PCA to take into account drop-out events and other technical bias. The displayed genes represent signatures or variable genes in respective gene sets.

### Clustering based on stem genes

To cluster the single-cell data based on stem cell state we employed a list of published of stem cell markers. Clustering was performed based on quantile-normalized log2(TPM) with pseudocounts of 1, followed by hierarchical clustering with ‘average-linking’ neighbor joining. Next we employed cutreeDynamic from WGCNA package (deepSplit = 1, MingroupSize = 20) to identify clusters within the resulting dendogram.

### Mapping of scRNA-seq data on TCGA

From TCGA portal we collected the HNSCC, Illumina HiSeq RNA-seq data and related clinical parameters (https://cancergenome.nih.gov). We specifically focused on data from cancer tissue and 5-years survival information which resulted in total 402 patients. The scaled expression value (tau values, calculated by RSEM) were extracted from the TCGA data and multiplied by 10^6^ to obtain transcripts per million (TPM). For each gene in TCGA data-set, the maximum mean gene expression was used for subsequent analysis. Next to molecular classification of TCGA dataset based on SOX2 and SOX9 expression (mean threshold of *SOX2* = 4.34 and *SOX9* = 4.29), 402 TCGA patients were grouped into four sub-classes (*SOX2*+/*SOX9−SOX2*+/*SOX9*+; *SOX2/SOX9*+ and *SOX2−*/SOX9*−*). Next, we analyzed the differential gene expression between these groups based on DEseq2 followed by generation of the survival curves for each of the sub-class.

### Statistical analysis

All graphs and statistical calculations were generated using Prism7 (GraphPad) software. Statistical significance was computed with the test indicated in each figure legend. The number of experiments and animals analysed are indicated in each figure.

## Electronic supplementary material

Supplementary Information

Peer Review File

Description of Additional Supplementary Files

Reporting Summary

Supplementary Data 1

Supplementary Data 2

Supplementary Data 3

Supplementary Data 4

Supplementary Data 5

## Data Availability

The Single cell RNA-seq datasets is available in the Gene Expression Omnibus repository, with the series record GSE117872 and ChIP-seq data is available at GSE120634. The authors declare that all data supporting the findings of this study are available within the manuscript and/or its Supplementary Files or are available from the corresponding authors upon request.
