# Supplementary material for: Longitudinal single-cell RNA sequencing of patient-derived primary cells reveals drug-induced infidelity in stem cell hierarchy
Source: Nat Commun. 2018 Nov 22;9:4931. doi: 10.1038/s41467-018-07261-3 (PMC6250721; doi:10.1038/s41467-018-07261-3)
Supplement: Supplementary file 1 — Supplementary Information [file 41467_2018_7261_MOESM1_ESM.pdf]

Longitudinal single-cell RNA sequencing of patient-derived primary cells reveals drug-induced infidelity in stem cell hierarchy

Sharma et al.

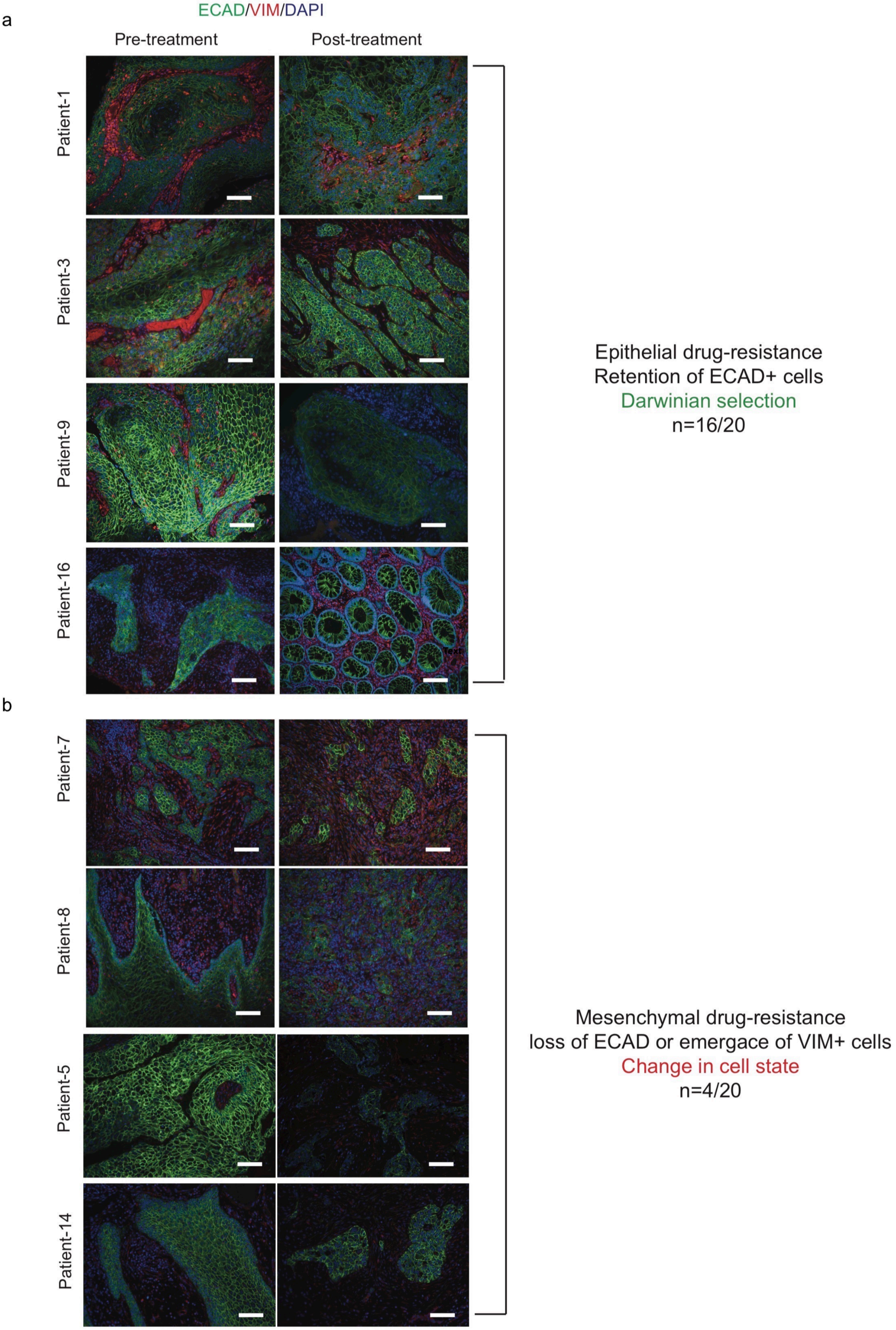
a
ECAD/VIM/DAPI
Pre-treatment
Post-treatment
Patient-1
Patient-3
Patient-9
Patient-16
Epithelial drug-resistance
Retention of ECAD+ cells
Darwinian selection
n=16/20
b
Patient-7
Patient-8
Patient-5
Patient-14
Mesenchymal drug-resistance
loss of ECAD or emergace of VIM+ cells
Change in cell state
n=4/20

Supplementary Figure 1 | Immunofluorescence-based investigation of drug-induced phenotypic evolution in OSCC patients. Protein expression analyses of epithelial (ECAD in green) and mesenchymal (VIM in red) markers in OSCC tumors (pre- and post-cisplatin treatment). **(a)** Drug-induced selection/retention of epithelial characteristic (Darwinian selection) and **(b)** cisplatin-induced *de novo* emergence of Vimentin expressing mesenchymal cells. Scale bar = 100μM.

Supplementary Figure 2

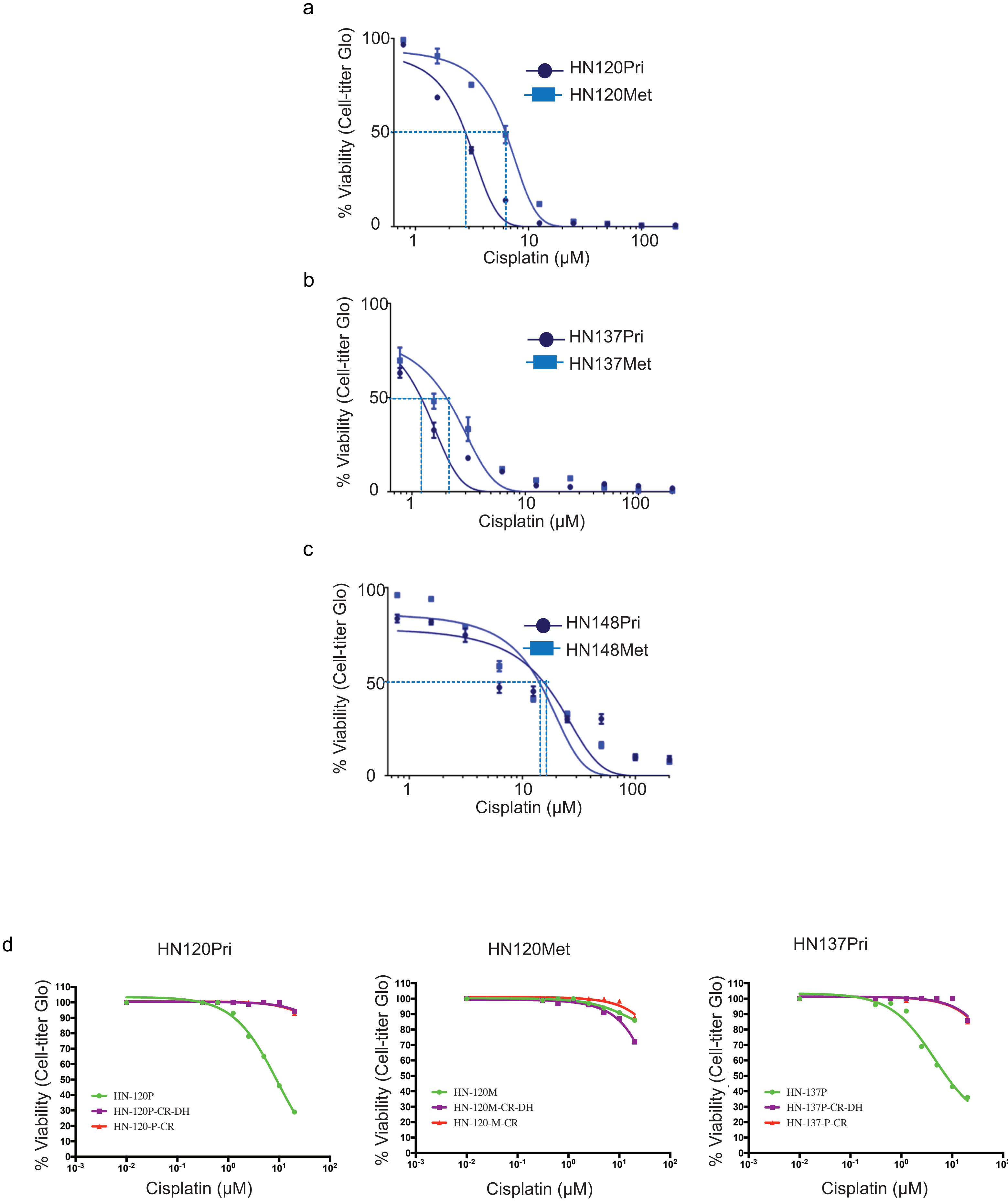

Supplementary Figure 2 | Generation of cisplatin resistant and drug holiday models of OSCC-PDPCs. (a-d) Determination of cisplatin IC_{50} value in primary and metastatic **(a)** HN120 and **(b)** HN137 cells and **(c)** HN148 cells. **(d)** Validation of the cytotoxic effect of cisplatin on naïve, drug-resistant and drug-holiday models of OSCC-PDPC (n=3, mean $\pm$ s.e.m.).

Supplementary Figure 3

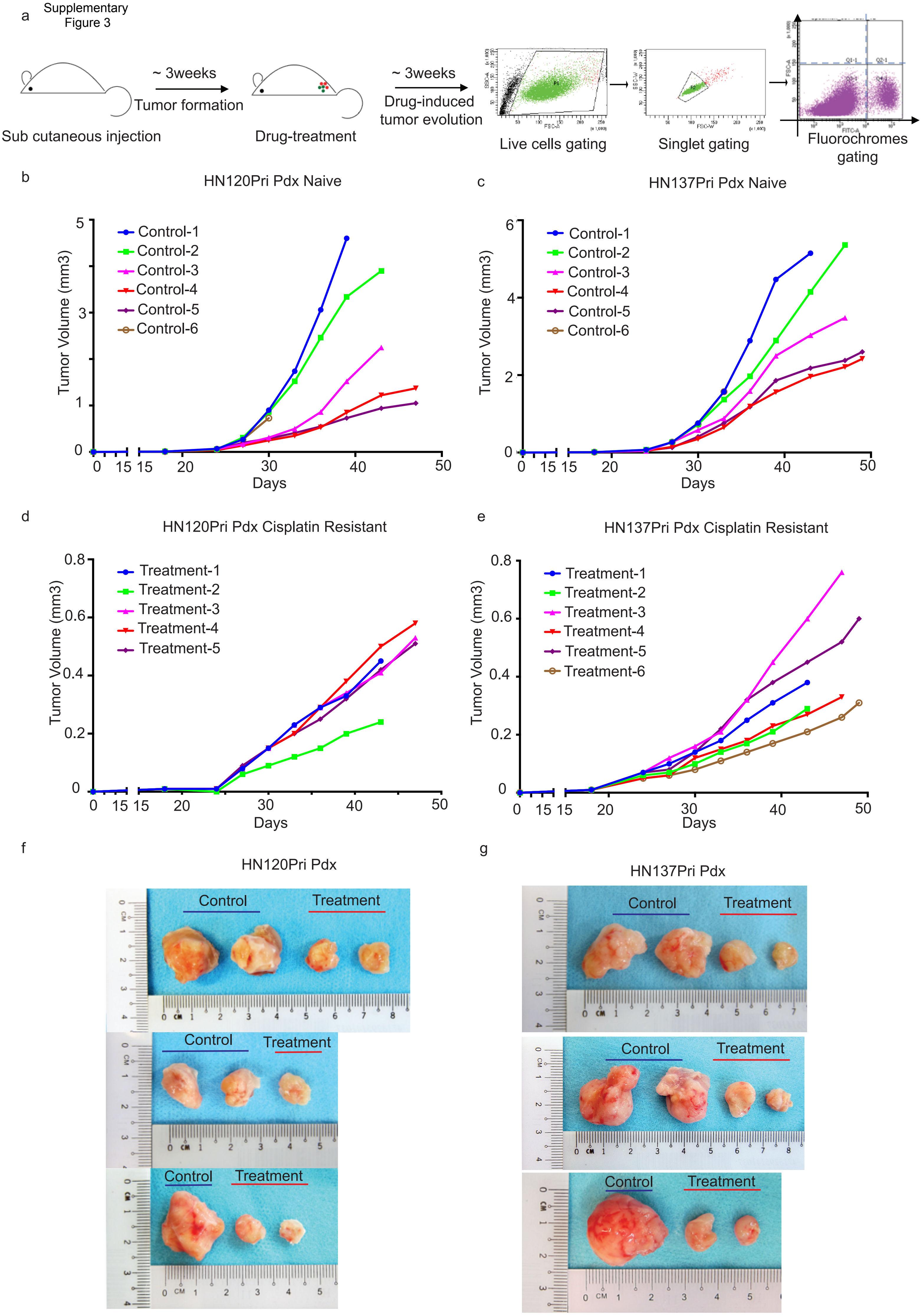

Supplementary Figure 3 | Generation of cisplatin resistant and drug holiday OSCC-Pdx models. (a-g) Generation of *in vivo* cisplatin-resistant Pdx models. **(a)** Schematic of experimental procedure and FACS gating strategy. Tumor growth in control **(b, c)** and cisplatin-treated **(d, e)** HN120Pri and HN137Pri Pdx models (n=5-6 mice per condition). Images of endpoint tumor isolation from control and cisplatin-treated HN120Pri **(f)** and HN137Pri **(g)** Pdx models (scale for reference).

Supplementary Figure 4

a

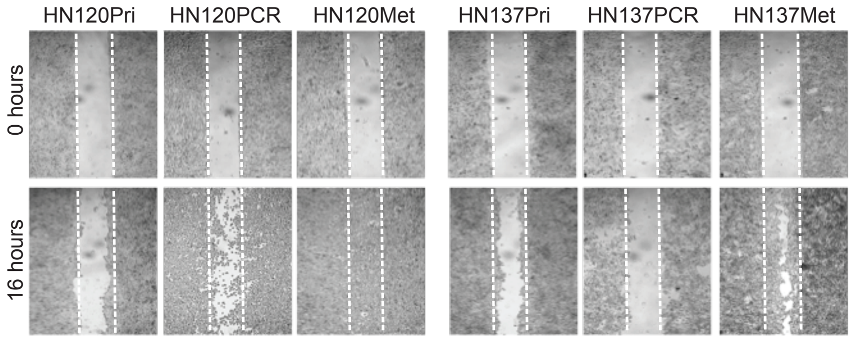

b

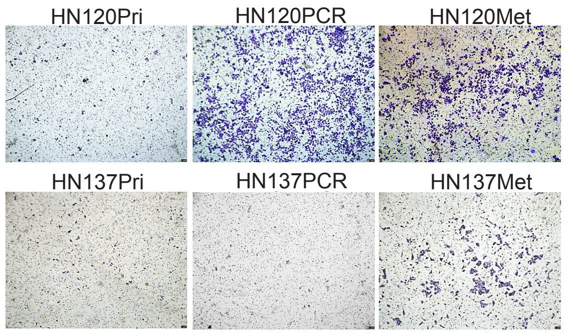

c

PDPC model	Lung metastasis (60 days post tail-vain injection)
HN120Pri	0/5
HN120PCR	3/5
HN120Met	4/5
HN120MCR	4/5

Supplementary Figure 4 | *In vitro* and *in vivo* EMT-like properties of HN120 naïve, drug-resistant and metastatic cells. Functional characterization of drug resistant HN137PCR and HN120PCR cells, compared to the parental and synchronous metastatic cell lines. *In vitro* cell migration (scratch-test) **(a)** and invasion (Boyden's chamber) assays **(b)** in treatment-naïve and drug-resistant cells from HN120 and HN137 models. Note the gain in migratory and invasive phenotypes in HN120PCR correlated with the gain in VIM+ mesenchymal-like cells. HN137PCR in contrast, displayed even further reduction in invasive and/or migratory properties, correlating with the loss of VIM+, and the gain of ECAD+ cells. **(c)** *In vivo* tail vain injection corroborates the metastatic properties of HN120PCR and HN120Metatatic cells. Scale bar = 50μM (a-b).

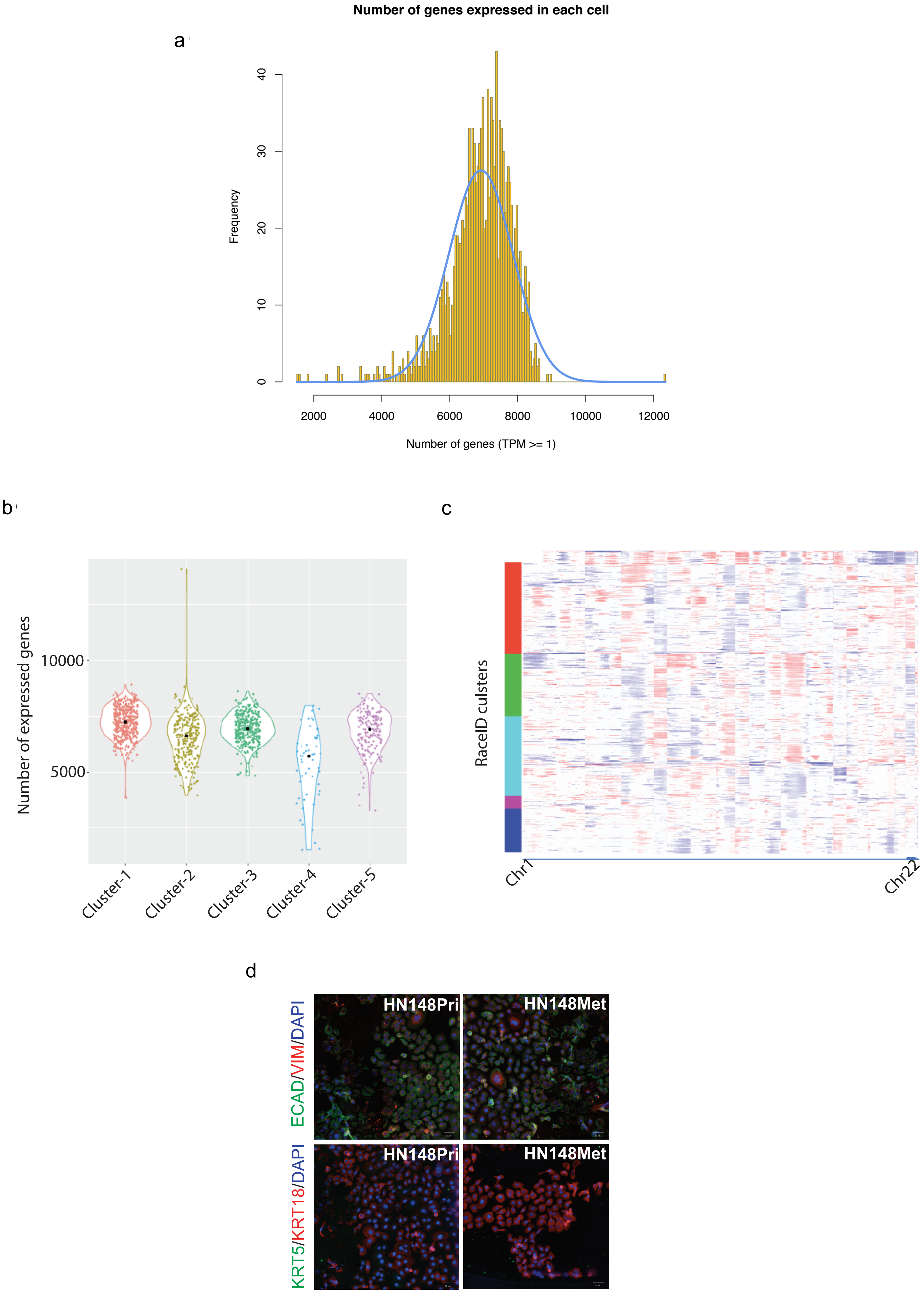
Number of genes expressed in each cell
a
Frequency
0
10
20
30
40
2000
4000
6000
8000
10000
12000
Number of genes (TPM >= 1)
b
Number of expressed genes
10000
5000
Cluster-1
Cluster-2
Cluster-3
Cluster-4
Cluster-5
c
RaceID culsters
Chr1
Chr22
d
ECAD/VIM/DAPI
KRT5/KRT18/DAPI
HN148Pri
HN148Met
HN148Pri
HN148Met

Supplementary Figure 5 | (a) Distribution of total number of genes expressed by 1302 single-cell RNA-seq libraries. **(b)** Distribution of total number of genes expressed by five RACE-ID groups. **(c)** Distribution of copy number variations (CNVs) in five RACE-ID groups. **(d)** Immunofluorescence analysis of epithelial (ECAD), basal (KRT5), mesenchymal (VIM) and luminal (KRT18) proteins in HN148 PDPC models (Scale bar = 50μM).

Supplementary Figure 6

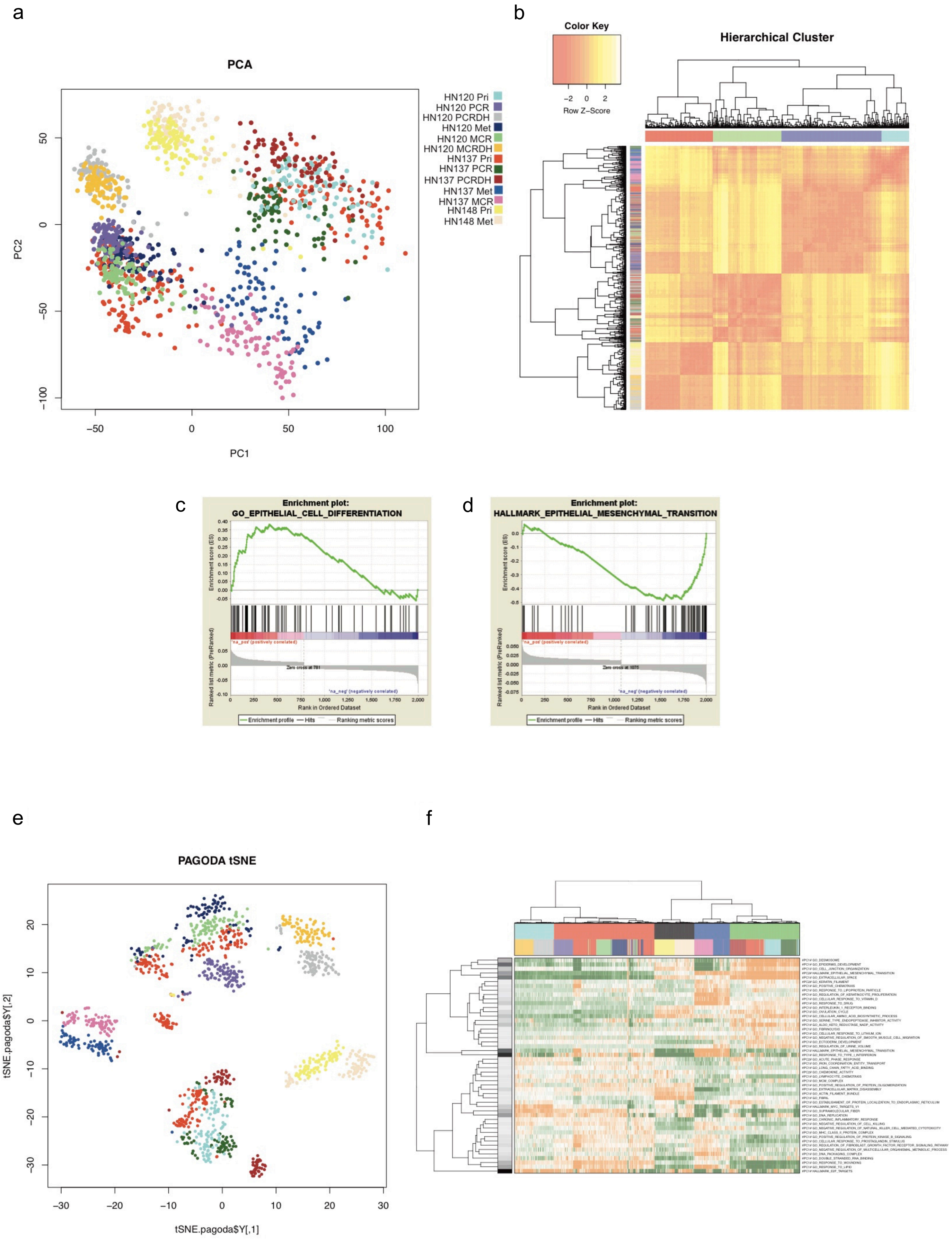

Supplementary Figure 6 | Principle component analysis (PCA) of single-cell RNA-seq libraries. PCA of various OSCC-PDCs and their drug-resistant models **(a)** Distribution and **(b)** hierarchical clustering of cells based on PC1 and PC2. Gene Ontology (GO) based enrichment plots for **(c)** PC1 (epithelial differentiation) and **(d)** PC2 (EMT). Visualization of PAGODA clustering **(e)** tSNE and **(f)** hierarchical clustering based on GO terms.

Supplementary Figure 7

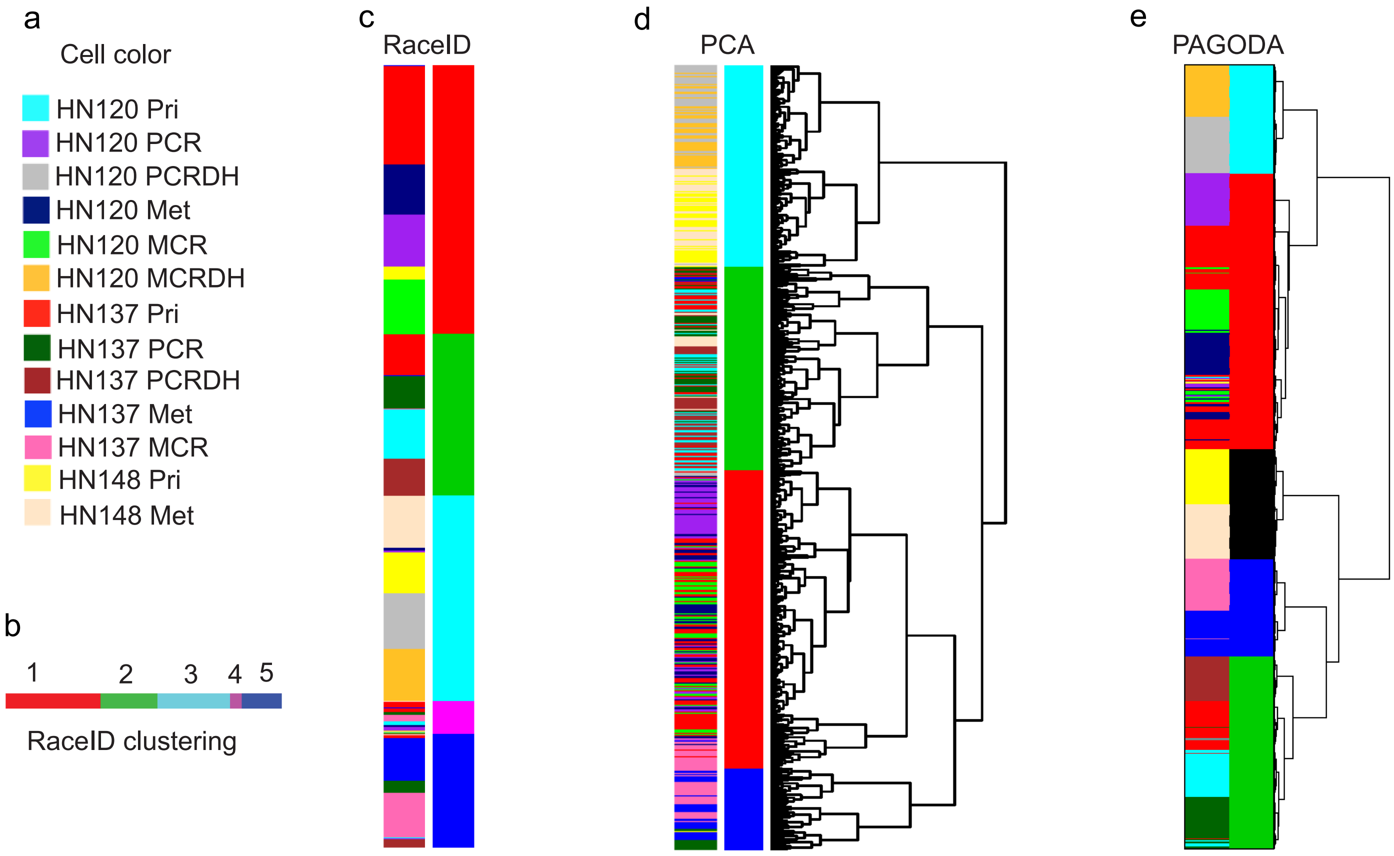

Supplementary Figure 7 | Comparison of three single cell analysis algorithms. (a) Cell ID color representing different OSCC-PDCs and their drug-resistant/holiday models. **(b)** Cluster identities determined by RaceID. **(c-e)** Concordance of cell clustering across 3 different algorithms, as shown in **(c)** RaceID, **(d)** PCA and **(e)** PAGODA.

Supplementary Figure 8

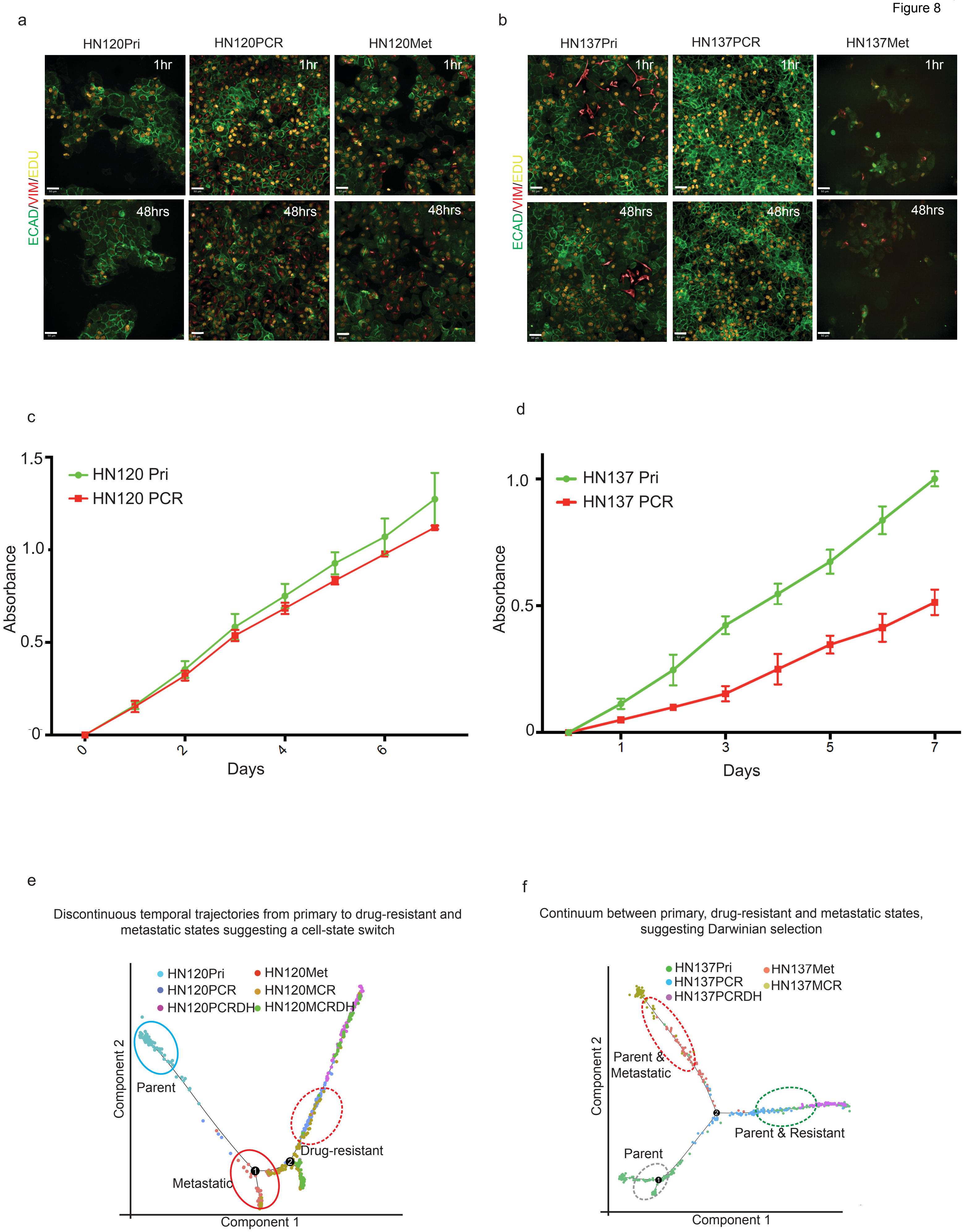

Supplementary Figure 8 | (**a-b)** EdU-based pulse-chase labeling assay to characterize the proliferative state of epithelial and mesenchymal OSCC-PDC cells. EdU labeling (yellow) was conducted for 1 hour to determine the numbers of actively proliferating cells, followed by 48 hours of chase to identify label-retaining quiescent cells. Epithelial and mesenchymal status of cells was determined by ECAD (green) and VIM (red) expression respectively in **(a)** HN120 and **(b)** HN137 cells. **(c-d)** Cell growth curves of **(c)** HN120Pri, HN120PCR and **(d)** HN137Pri, HN137PCR cells (n=3, mean $\pm$ s.e.m.). **(e-f)** Pseudo-temporal ordering of HN120 (e) and HN137 (f) naïve, drug-resistant and drug-holiday models. Please note the uncoupling/discontinuity of cell states for HN120 suggesting cellular-reprogramming; whereas HN137 represents a continuum of states suggesting clonal selection. Scale bar = 50μM (a-b).

Supplementary Figure 9

Drug-induced loss-of SOX2+ cells and gain-of SOX9+ cells in HN120 model

a

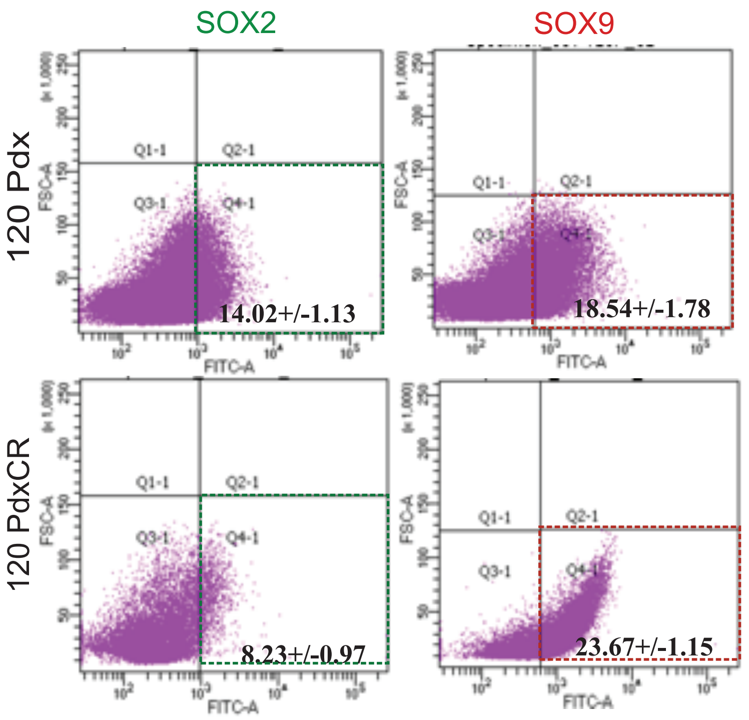

Drug-induced gain-of SOX2+ cells in HN120 model

b

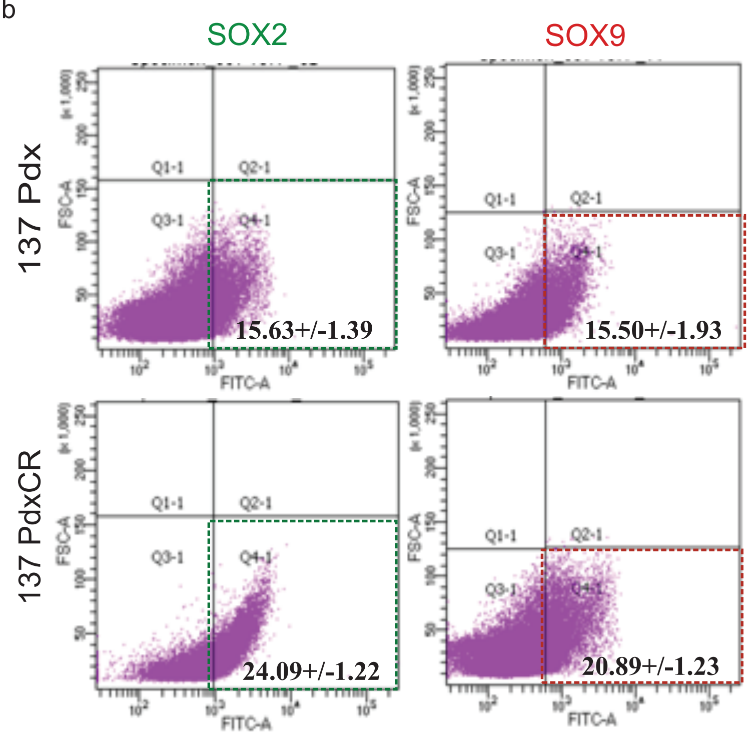

c

Higher expression of chromatin remodelers & Hes1 in Sox2+ HN120Pri cells

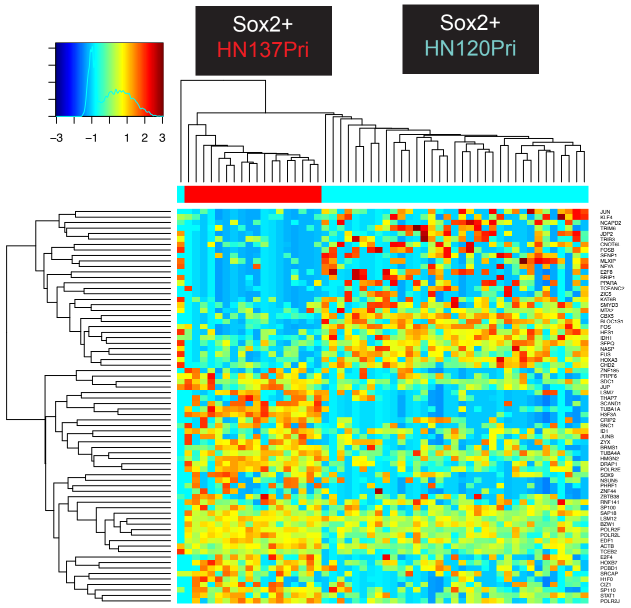

Supplementary Figure 9 | (a, b) Flow-cytometeric analysis of SOX2 and SOX9 positive cells reveals a reduction of SOX2+ population and the gain of SOX9+ cells in the HN120 Pdx models (a). Cisplatin-induced selection of pre-existing SOX2+ cancer stem-like cells in the HN137 model (b) (n=5-6 mice per condition). **(c)** Differential gene expression profile between Sox2 expressing HN120 and HN137 primary cells.

Supplementray Figure 10

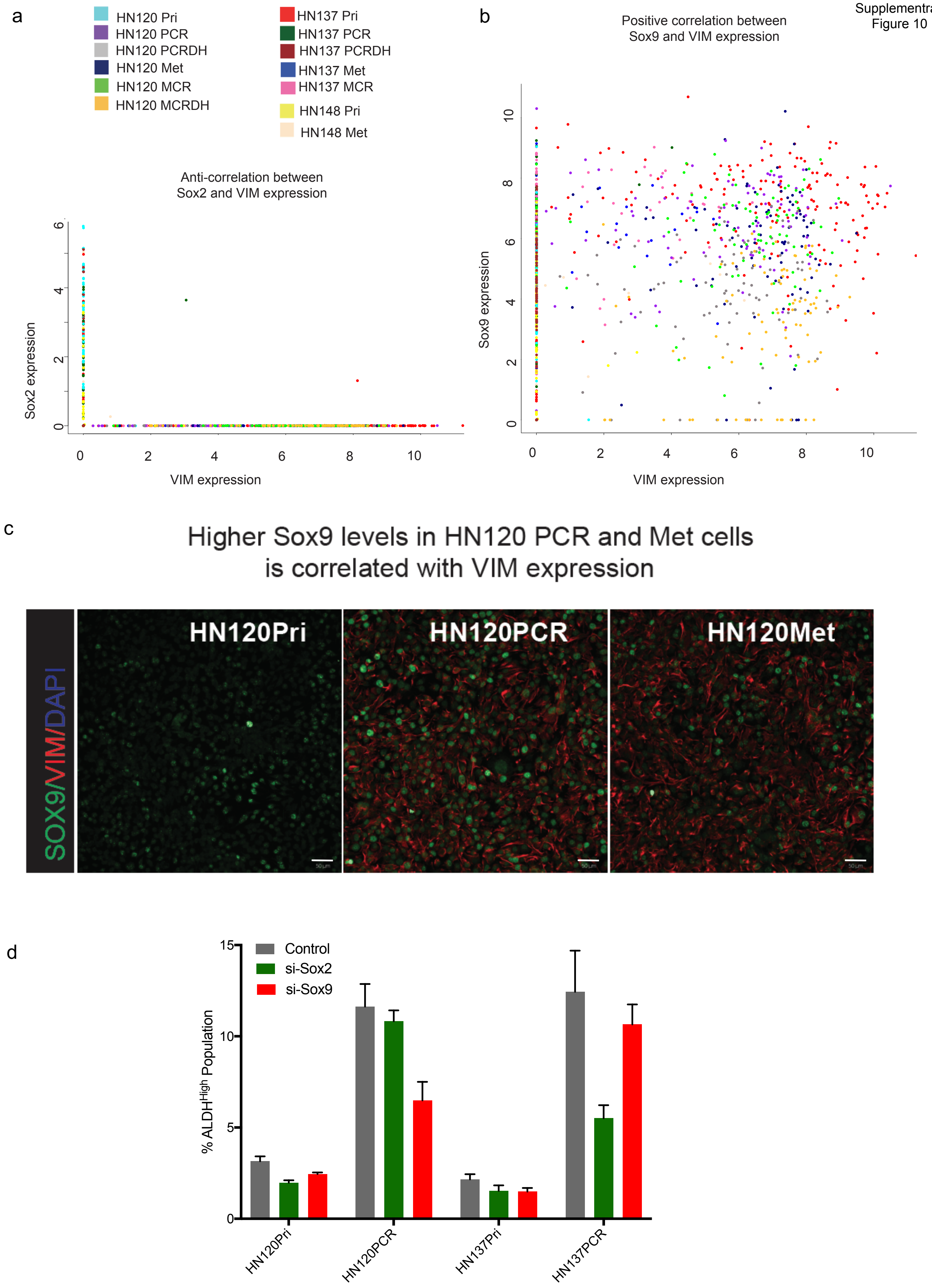

Supplementary Figure 10 | Interplay of Sox2 and Sox9 in maintaining cell fates. (a, b) Correlation plot for co-expression of Sox2 (a) or Sox9 (b) with Vimentin in 1302 single-cell libraries from naïve, drug-resistant and drug-holiday models. Note the strong correlation between Sox9 and Vimentin expression, whereas a striking anti-correlation between Sox2 and Vimentin expression. **(c)** Immunofluorescence-based analysis of Sox9 (green) and Vimentin (red) expression in HN120 primary, drug-resistant and metastatic cells (Scale bar = 50μM). **(d)** Effect of siRNA-mediated down-regulation of Sox2 and Sox9 on ALDH+ cells in primary and drug resistant cells (n=3, mean ± s.e.m.).

Supplementary Figure 11

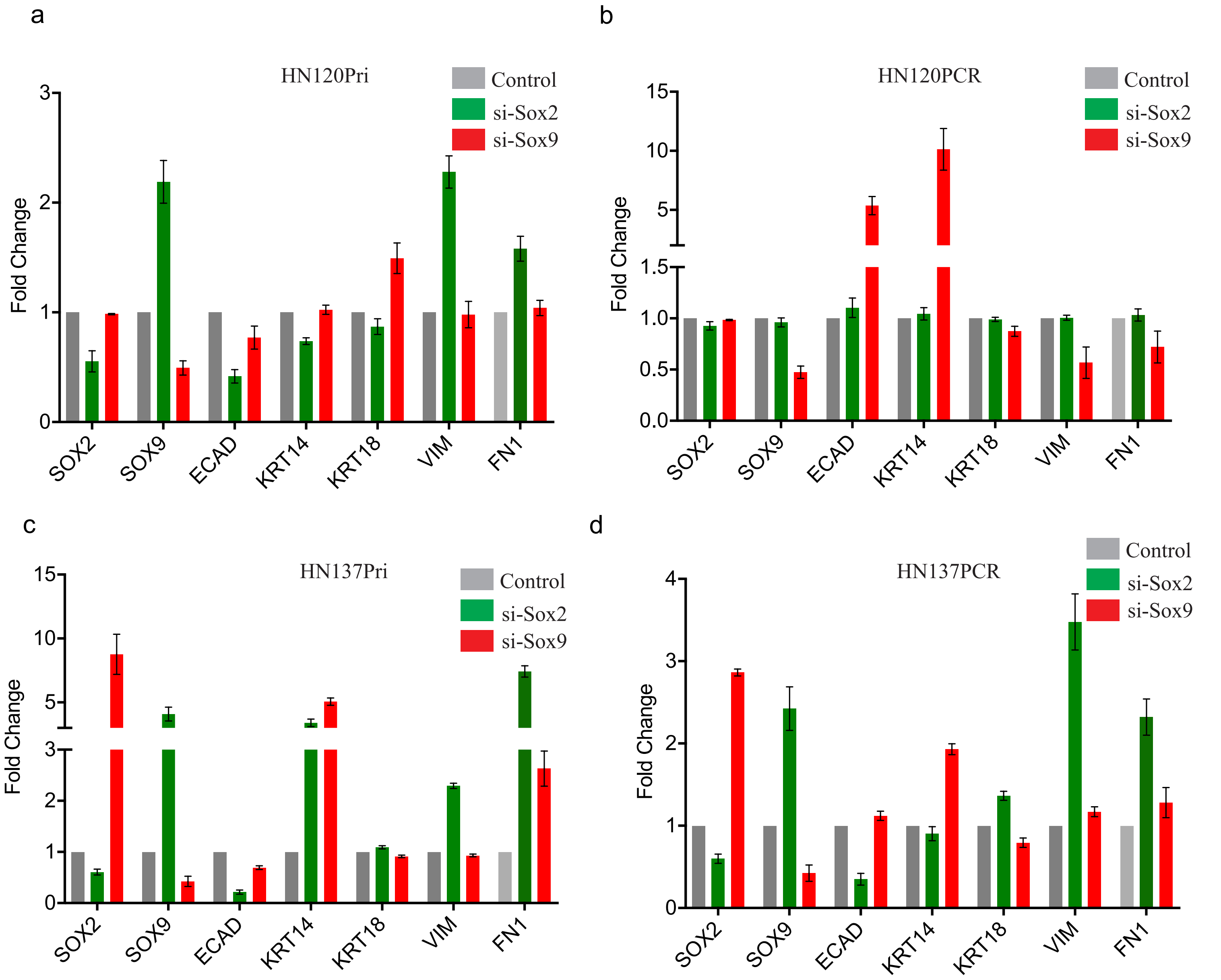

Supplementary Figure 11 | Effect of Sox2 and Sox9 loss-of-function on cell-state specific genes on (a) HN120 Pri, (b) HN120 PCR, (c) HN137Pri and (d) HN137PCR cells (n=3, mean ± s.e.m.).

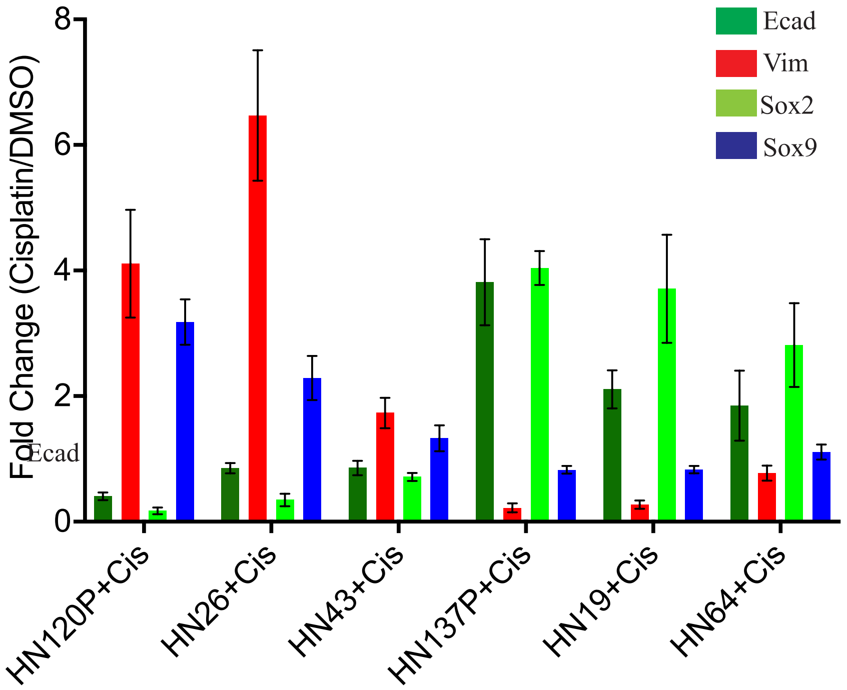
Fold Change (Cisplatin/DMSO)
8
6
4
2
0
Ecad
Ecad
Vim
Sox2
Sox9
HN120P+Cis
HN26+Cis
HN43+Cis
HN137P+Cis
HN19+Cis
HN64+Cis

Supplementary Figure 12 | Effect of cisplatin on cell- and stem-state specific genes in different patient derived primary culture of OSCCs (n=3, mean ± s.e.m.).

Supplementary Figure 13

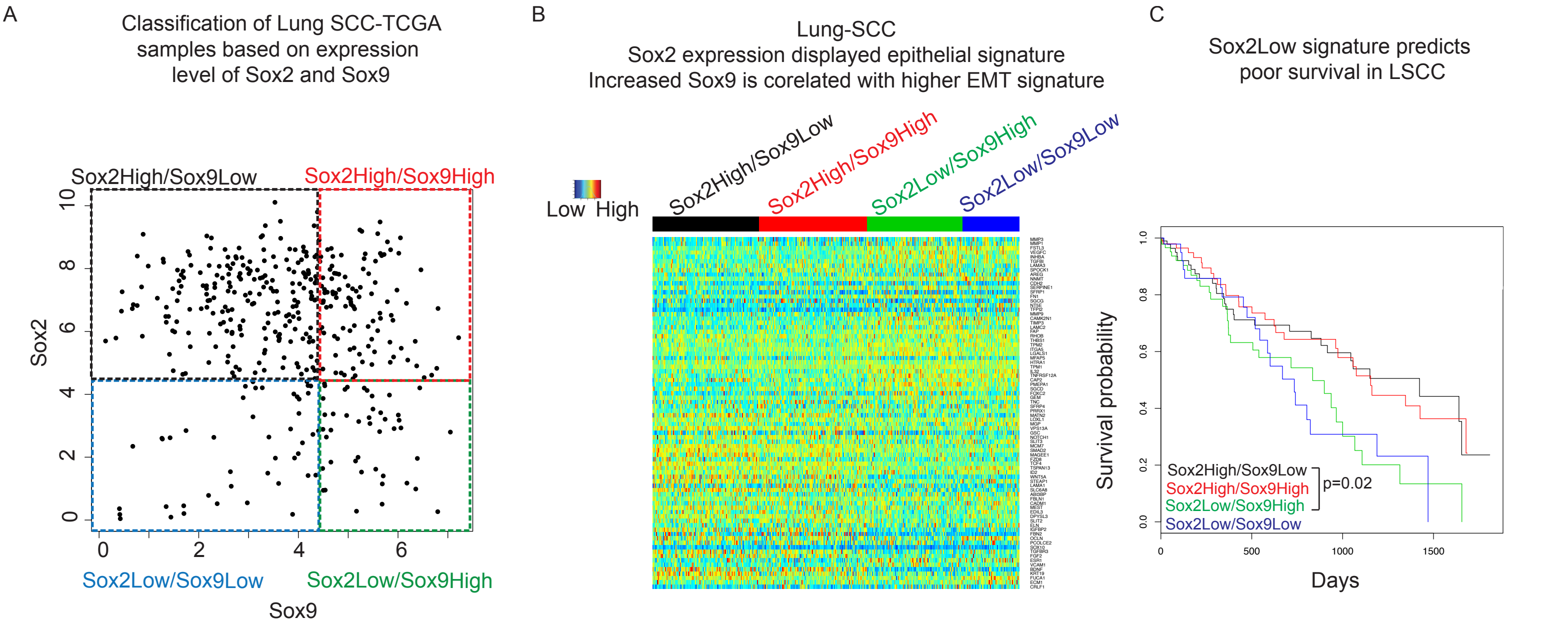

Supplementary Figure 13 | Sox2 and Sox9 based molecular subtyping of lung squamous cell carcinoma. (a) Virtual sorting of TCGA Lung-SCCs in Sox2High/Sox9Low, Sox2High/Sox9High, Sox2Low/Sox9High and Sox2Low/Sox9Low sub-sets **(b)** Differential expression of EMT markers in sub-sets based on the “sorted” Sox2 and Sox9 levels and **(c)** disease-free survival based on gene-expression profiles (Cox regression *P* value <0.05). Note: Detailed statistical analysis presented in Supplementary Table 3.

a

Gain-of H3K27ac and loss-of H3K27me3 marks on EMT assocaited Vimentin promoter in HN120PCR and HN120Met cells

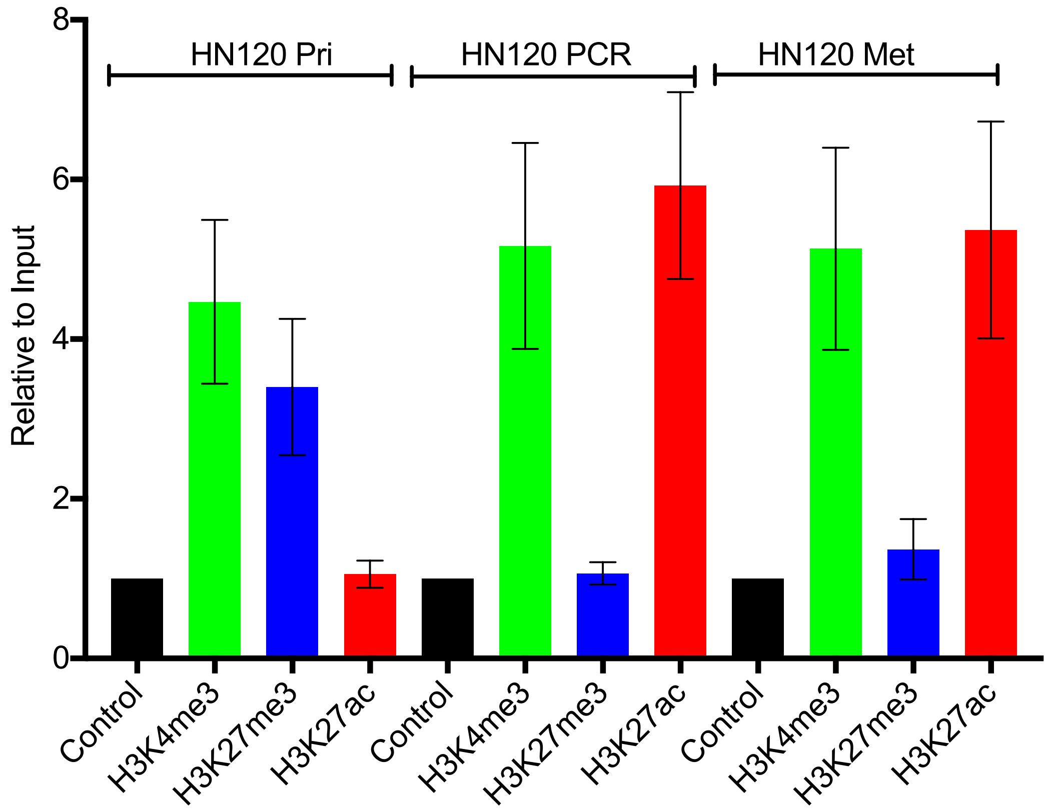

b

Gain-of H3K27ac/H3K4me3 ratio on cellular-plasticity (CP) specific promoters in HN120PCR and HN120PCRDH models

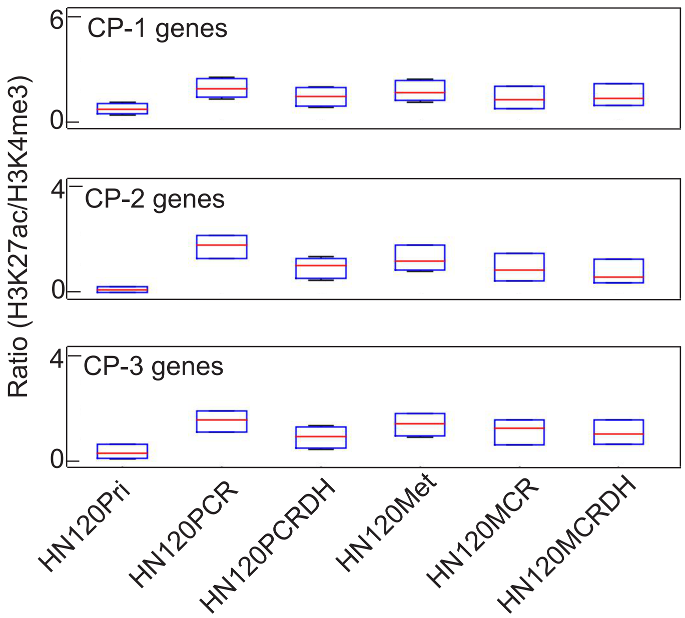

Supplementary Figure 14 | (a) ChIP-qPCR analysis of the repressive chromatin mark H3K27me3 on vimentin promoter in HN120Pri, HN120PCR and HN120 Met cells (n=3, mean $\pm$ s.e.m.). **(b)** Ratio of H3K27ac/H3K4me3 chromatin modifications on cellular-plasticity (CP) specific promoters in HN120 models.

Supplementary Figure 15

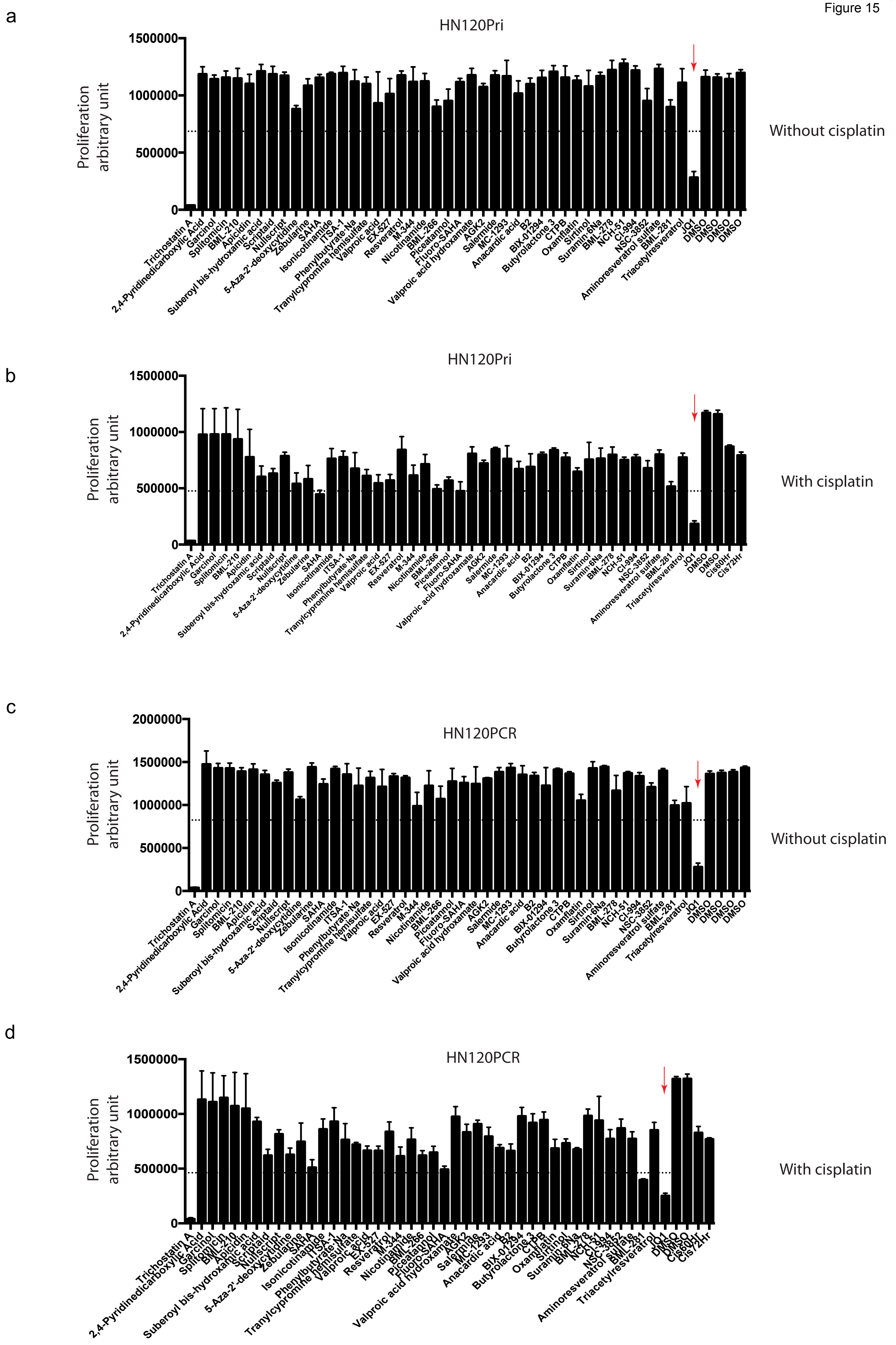

Supplementary Figure 15 | Small molecule screens to identify drugs that display synthetic lethality with cisplatin. Effect of small molecule chromatin remodelers on cell viability of naïve HN120 primary and their drug-resistant models either in the absence **(a, c)** or presence **(b, d)** of cisplatin (n=4, mean $\pm$ s.d.).

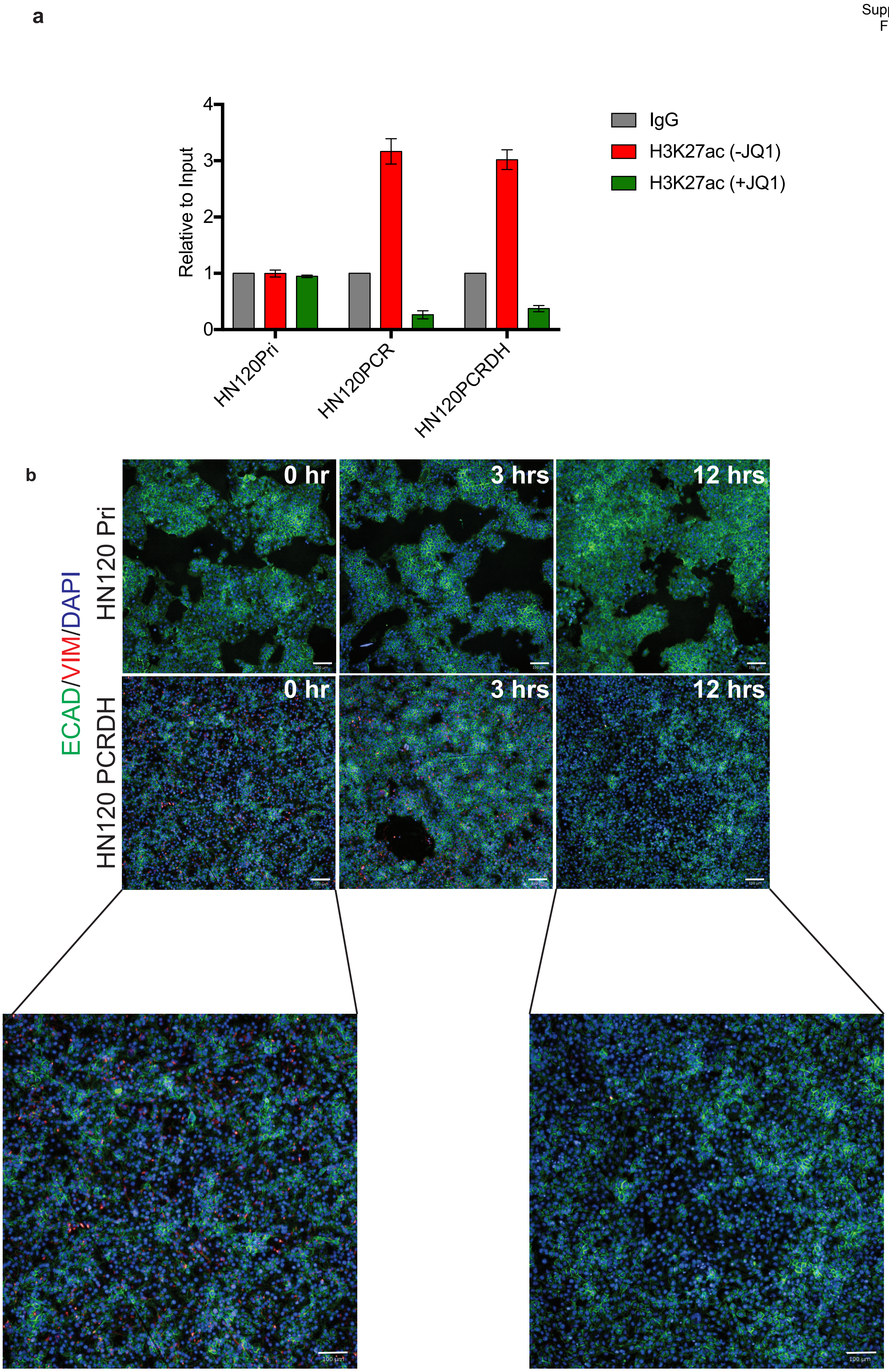
a
4
3
2
1
0
Relative to Input
HN120Pri
HN120PCR
HN120PCRDH
IgG
H3K27ac (-JQ1)
H3K27ac (+JQ1)
b
0 hr
3 hrs
12 hrs
0 hr
3 hrs
12 hrs
HN120 Pri
HN120 PCRDH
ECAD/VIM/DAPI
100 µm
100 µm

Supplementary Figure 16 | Effect of the chromatin remodeler JQ1 on cellular reprogramming. (a) ChIP-qPCR for H3K27ac signal on Vimentin promoter from HN120 naïve, drug resistant and holiday cells in the presence or absence of JQ1 (n=3, mean $\pm$ s.e.m.). **(b)** Immunofluorescence for ECAD (green) and VIM (red) expression in the presence of JQ1 at 0, 3, and 12hr time points Scale bar = 100μM.

PDC model	HN120Pri	HN120Met	HN137Pri	HN137Met	HN148Pri	HN148Met
**Naïve**	2.78	6.31	1.6	2.32	12.26	12.72
**Replicate 1**	17.23	20.75	13.15	12.27	NA	NA
**Replicate 2**	17.77	15.95	17.11	14.76	NA	NA
**Replicate 3**	17.42	16.14	15.76	14.14	NA	NA
**Replicate 4**	16.31	NA	15.67	NA	NA	NA

Supplementary Table 1: Cisplatin IC50 value of OSCC PDPC models and their drug-resistant clones

Primer	Sequence 5'- 3'	Application
**Vimentin Promoter Fwd**	CCGCAGCCCCGAGACCGCCGCGCA	ChIP qPCR
**Vimentin Promoter Rev**	GTCCCGTTACTTCAGCGCTGGGCT	ChIP qPCR
**SOX2 Fwd**	CCCCCGGCGGCAATAGCA	cDNA qPCR
**SOX2 Rev**	TCGGCGCCGGGGACATACAT	cDNA qPCR
**SOX9 Fwd**	GGGAAGGCCGCCCAGGGCGA	cDNA qPCR
**SOX9 Rev**	TGCCTTGCCCGACTGCAGTTCT	cDNA qPCR
**ECAD Fwd**	TGCCCAGAAAATGAAAAAGG	cDNA qPCR
**ECAD Rev**	GTGTATGTGGCAATGCGTTC	cDNA qPCR
**KRT14 Fwd**	TTCTGAACGAGATGCGTGAC	cDNA qPCR
**KRT14 Rev**	GCAGCTCAATCTCCAGGTTC	cDNA qPCR
**KRT18 Fwd**	CACAGTCTGCTGAGGTTGGA	cDNA qPCR
**KRT18 Rev**	GAGCTGCTCCATCTGTAGGG	cDNA qPCR
**VIM Fwd**	GAGAACTTTGCCGTTGAAGC	cDNA qPCR
**VIM Rev**	TCCAGCAGCTTCCTGTAGGT	cDNA qPCR
**FN1 Fwd**	CAGTGGGAGACCTCGAGAAG	cDNA qPCR
**FN1 Rev**	TCCCTCGGAACATCAGAAAC	cDNA qPCR
**B2m Fwd**	CCTGAATTGCTATGTGTCT	cDNA qPCR
**B2m Rev**	TGATGCTGCTTACATGTCT	cDNA qPCR

Supplementary Table 2: Primer sequence used in the study

Mean cut-off values	HNSCC	Lung-SCC
**SOX2**	4.293612	5.988543
**SOX9**	4.265253	3.79839

P-values (5 year survival plots)

	HNSCC	Lung-SCC
**Sox2High;Sox9Low vs Sox2High;Sox9High**	0.160588	0.98626
**Sox2High;Sox9Low vs Sox2Low;Sox9High**	0.043547	0.028182
**Sox2High;Sox9Low vs Sox2Low;Sox9Low**	0.292037	0.053203
**Sox2High;Sox9High vs Sox2Low;Sox9High**	0.365362	0.014471
**Sox2High;Sox9High vs Sox2Low;Sox9Low**	0.883038	0.064218
**Sox2Low;Sox9High vs Sox2Low;Sox9Low**	0.303778	0.971737

Supplementary Table 3: Mean cut-off value for determining Sox2 and Sox9 high vs low tumors in TCGA dataset and P-value for 5-year survival plots based on Sox2 and Sox9 expression in HNSCC (Figure 5g-i) and Lung-SCC (Supplementary Figure 13) TCGA datasets.
