# Supplementary material for: Longitudinal single-cell RNA sequencing of patient-derived primary cells reveals drug-induced infidelity in stem cell hierarchy
Source: Nat Commun. 2018 Nov 22;9:4931. doi: 10.1038/s41467-018-07261-3 (PMC6250721; doi:10.1038/s41467-018-07261-3)
Supplement: Supplementary file 3 — Description of Additional Supplementary Files [file 41467_2018_7261_MOESM3_ESM.pdf]

Description of Additional Supplementary Files

Supplementary Data 1: Clustering of 1302 single-cell RNA-seq libraries representing various models

Supplementary Data 2: Gene expression profile of different RaceID clusters

Supplementary Data 3: Genes representing top-6 PCs from PCA based analysis

Supplementary Data 4: List of stemness related genes

Supplementary Data 5: List of differentially upregulated genes in HN120PCR compared to HN120Pri cells (for clustering H3K4me3 marks on promoters)
