# Supplementary material for: Longitudinal single-cell RNA sequencing of patient-derived primary cells reveals drug-induced infidelity in stem cell hierarchy
Source: Nat Commun. 2018 Nov 22;9:4931. doi: 10.1038/s41467-018-07261-3 (PMC6250721; doi:10.1038/s41467-018-07261-3)
Supplement: Supplementary file 2 — Peer Review File [file 41467_2018_7261_MOESM2_ESM.pdf]

Editorial Note: this manuscript has been previously reviewed at another journal that is not operating a transparent peer review scheme. This document only contains reviewer comments and rebuttal letters for versions considered at Nature Communications

Reviewer #1 (Remarks to the Author):

The authors have addressed all of my concerns. I am satisfied with it.

Reviewer #3 (Remarks to the Author):

I've now read this revised manuscript and reviewed the manner in which the authors have responded to the prior criticisms raised. In my view, the authors have very thoughtfully commented on and rectified the prior issues raised. I find the revised paper well-suited to Nature Communications and recommend publication.
